# Universal Polaronic Behavior in Elemental Doping of MoS_2_ from First-Principles

**DOI:** 10.1021/acsnano.4c08366

**Published:** 2024-12-02

**Authors:** Soungmin Bae, Ibuki Miyamoto, Shin Kiyohara, Yu Kumagai

**Affiliations:** Institute for Materials Research, Tohoku University, 2-1-1 Katahira, Aoba-ku, Sendai 980-8577, Japan

**Keywords:** 2D materials, transition metal dichalcogenides, MoS_2_, defects, doping, density functional theory

## Abstract

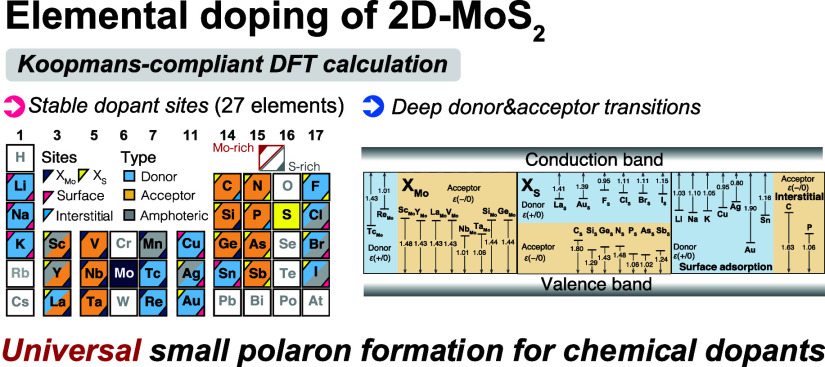

Elemental doping of two-dimensional (2D) semiconductors is crucial for manipulating their electrical and optical properties and enhancing the performance of advanced 2D devices. However, doping methods, such as ion implantation and chemical vapor deposition, can produce various outcomes extensively, depending on the chemical environment. We systematically study the elemental doping of the monolayer MoS_2_ by using density-functional theory calculations, which identify thermally stable sites among atomic substitutions, surface adsorption, and lattice interstitials of 27 elemental dopants, along with their formation energies and charge transition levels. By adopting the Koopmans-compliant hybrid functionals, the hydrogenic states predicted by semilocal functionals transform into localized polaronic states, which universally exhibit deep transitions located 1.0 eV away from the band edges. This polaronic behavior persists even in bulk MoS_2_, which suggests impurity conduction as the predominant carrier conduction mechanism. Our study offers fundamental insights into elemental doping in MoS_2_, which could be essential for doping transition metal dichalcogenides and similar 2D semiconductors.

## Introduction

Two-dimensional (2D) semiconductors are attracting increasing attention with the hope of achieving breakthroughs in higher performance and further miniaturization beyond conventional three-dimensional (3D) semiconductors. For its applications in various research areas, elemental doping has now become a key technology. Doping of 2D semiconductors is able to extensively modulate carrier conductivity,^1−4^ optical properties (light absorption and luminescence),^5−7^ catalysis behavior,^8,9^ and superconductivity.^10,11^ Given their significant role, a tremendous number of investigations from both experimental and theoretical studies have been conducted.

While a wide variety of 2D materials have been suggested and synthesized, transition metal dichalcogenides (TMDs) stand out as the most established class of 2D semiconductors. They have been successfully fabricated using both physical exfoliation and chemical synthesis techniques and effectively integrated into the operation of electronic and optical devices.^12−14^ Doping TMDs has been widely performed with various experimental processes, including ion implantation,^15,16^ plasma treatment,^17,18^ surface adsorption,^1,19^ and chemical reactions.^4,20^ However, optimizing doping conditions to enhance the electrical conductivity and carrier density remains an active area of research. Particularly, in doped 2D TMDs, impurity conduction is considered the primary mechanism for carrier transport in doped TMDs.^4,15,21^

The elemental doping in TMDs has been extensively explored through theoretical calculations based on density-functional theory (DFT).^22−26^ The DFT simulations, being idealized and simplified compared to experimental conditions, can reveal the intrinsic properties of dopants without the extrinsic influences caused by varying experimental conditions such as the substrate and lattice strain. While many DFT studies have provided numerical predictions, previous calculations often suffer from both qualitative and quantitative limitations: (i) errors caused by finite model sizes in total energy calculations of charged defects; (ii) self-interaction errors in semilocal exchange–correlation functionals, leading to spurious delocalization of carriers; and (iii) limited configurations of dopant sites. These main challenges have been addressed by adopting (i) *a posteriori* image-charge corrections;^24,26−28^ (ii) hybrid functionals or the GW approximation;^29−31^ and (iii) a comprehensive consideration of various atomic configurations, including surface and interstitial sites.^25,32^ Several studies have partially overcome these challenges, yet not fully; for instance, DFT calculations employing the hybrid functionals can greatly minimize the self-interaction errors but are restricted to a few intrinsic vacancies and selected dopants due to high computational costs.^25,30,31^

In this work, we present a comprehensive DFT study of the elemental doping in MoS_2_ that is a prototype of 2D TMDs. The monolayer (ML) MoS_2_ can be synthesized via physical exfoliation,^33,34^ chemical vapor deposition,^2,4^ or alternative fabrication techniques,^35,36^ finding extensive use in applications such as field-electric transistors, photodetectors, sensors, and spintronic devices.^12,13^ Although Re^37−39^ and Nb^4,40,41^ have been identified as effective *n*-type and *p*-type dopants in experiments, respectively, further investigation of other dopants is necessary to extensively control various physical properties. Therefore, we aim to thoroughly investigate a wide variety of dopants.

Initially, the stability of 27 dopants in ML MoS_2_ at various lattice sites, including two substitutional sites (Mo site and S site), was established based on the semilocal PBEsol functional. We considered three surface adsorption sites and two interstitial sites under the extreme Mo-rich and S-rich chemical potential conditions, which determine the thermal ground state of dopants’ sites. The Koopmans-compliant hybrid functional fulfilling the generalized Koopman’s condition^42,43^ is adopted to ensure self-interaction-free calculations for the donors and acceptors. While the PBEsol functional exhibits delocalized hydrogen-like states for several dopants, the hybrid functionals reveal universal small polaron behavior across all donors and acceptors with their transition levels being 1.0 eV apart from the band edges. It has also been demonstrated that such polaronic states persist in bulk MoS_2_, which implies that the polaronic behavior can be preserved in the surrounding dielectric mediums, such as substrates or additional stacking layers. The universal formation of the small polarons with deep transition levels suggests that the primary carrier conduction of doped MoS_2_ is attributed to an impurity conduction mechanism.

## Results and Discussion

**Figure 1:**
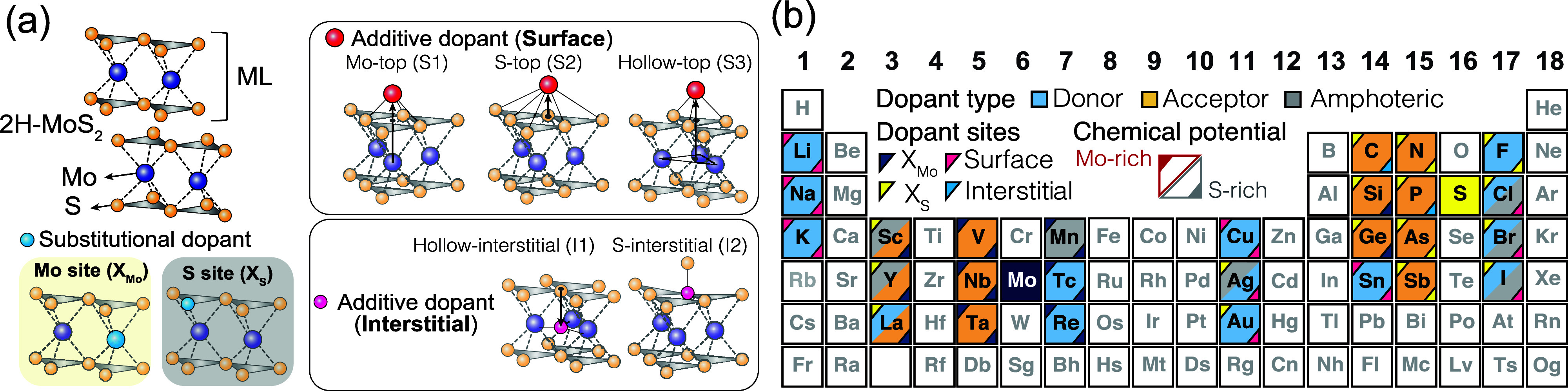
(a) Layered structure of 2H–MoS_2_ with substitutional, surface adsorption, and interstitial dopant sites. Bottom left panel: the substitutional Mo (X_Mo_) and S (X_S_) sites are denoted. Top right panel: three surface adsorption sites, namely, Mo-top (S1), S-top (S2), and hollow-top (S3). Right bottom panel: two interstitial sites, including hollow-interstitial (I1) and S-interstitial (I2). (b) Periodic table denoting stable dopant sites and their doping types, classified as donor, acceptor, and amphoteric states. For Mo-rich and S-rich conditions, the ground stable sites are marked with labels at the top-left and bottom-right corners, respectively. Dopant types depending on the chemical potential are indicated by the filled colors of the blocks.

Among the existing polymorphs of MoS_2_, the trigonal prismatic phase (2H) is found to be thermodynamically stable.^13,44^ The crystal structure of 2H–MoS_2_ and possible dopant sites in ML MoS_2_ are illustrated in Figure 1a. Note that the band gap of MoS_2_ increases with the reduction in stacking layers, peaking at 2.7 eV with a transition to the direct gap upon reaching the ML limit.^12,45^ For 27 dopants, in addition to two substitutional sites (Mo site and S site), three surface adsorption and two interstitial sites in Figure 1a were comprehensively explored using the semilocal PBEsol functional.^46^ In this study, we distinguish between adsorption and interstitial sites, despite neither involving the removal of host atoms due to the anticipated energy barriers for migration between them. Therefore, we categorize the stable dopant sites into three groups, namely, substitutional sites, absorption sites, and interstitial sites. We determine the stable dopant sites at extreme Mo-rich and S-rich chemical potential limits. The results are summarized in the periodic table of Figure 1b, with details provided later.

**Figure 2:**
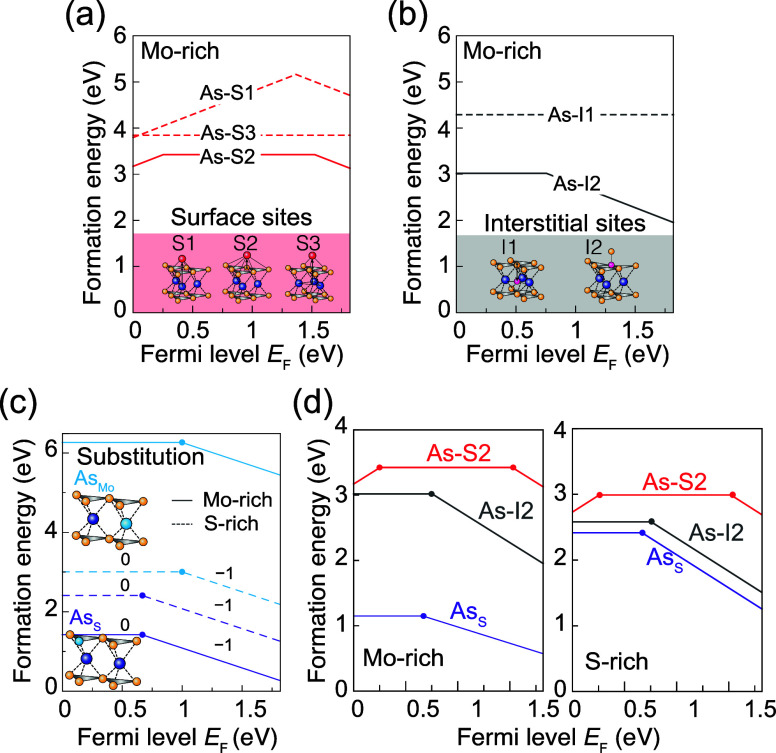
Formation energies of the As dopant in the monolayer MoS_2_ as a function of the Fermi level. (a) Formation energies of the As dopant residing on surface adsorption sites (S1, S2, and S3), (b) interstitial sites (I1 and I2), and substituted sites (As_Mo_ and As_S_). (a) and (b) consider the Mo-rich condition, while (c) considers both Mo-rich and S-rich conditions. The solid lines and dashed lines represent the formation energies in Mo-rich and S-rich conditions, respectively. (d) Formation energies of the As dopant at the stable sites among surface adsorption, lattice interstitials, and substitutional sites (S2, I2, and As_S_).

The procedure for identifying the stable dopant sites is outlined in Figure 2, using the arsenic (As) dopant as an example. The formation energies at (a) three surface adsorption sites (S1, S2, and S3) and (b) two interstitial sites (I1 and I2) are presented in Figure 2a,b. For each configuration, charge states from *q* = −2 to +2 are considered with the energy corrections for charged defects (see the Methods section). The stable sites S2 and I2 in surface adsorption and lattice interstitial groups are highlighted with solid lines. Figure 2c shows the formation energies of the substituted As dopants at the Mo site and the S site (As_Mo_ and As_S_). As_S_ is found to be stable compared to As_Mo_, regardless of the chemical potentials. Figure 2d shows the formation energies of the thermally stable configurations for four groups (S2, I2, and As_S_). Consequently, As_S_ is found to be the most stable site (referred to as the *ground state*, henceforth) for the As dopant in ML MoS_2_ over all possible configurations.

**Figure 3:**
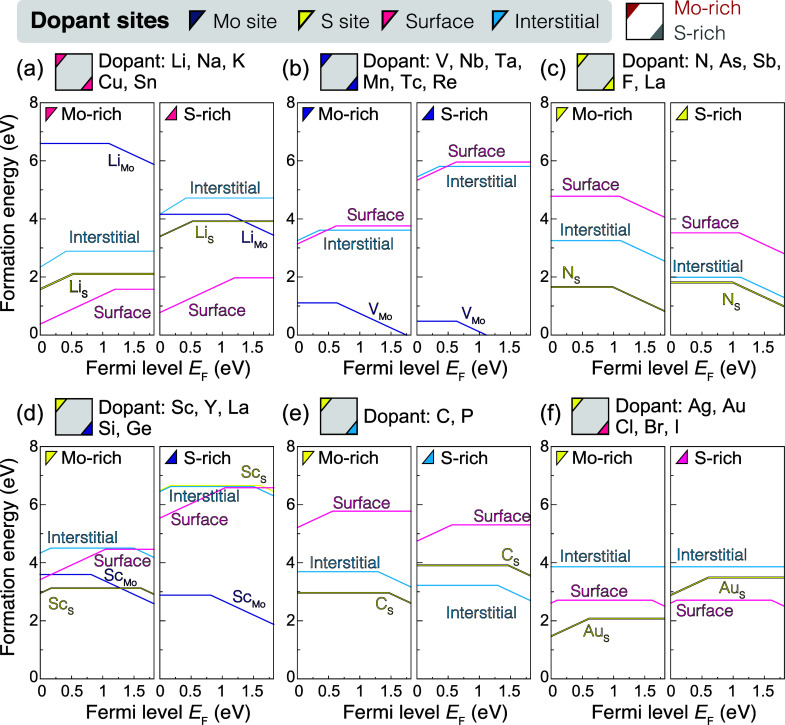
Classified 27 dopants into six categories based on the ground states under the Mo-rich and S-rich conditions: (a–c) (a) surface adsorptions, (b) Mo substitutions, (c) S substitutions are stable irrelevant to the chemical potentials. (d–f) (d) S substitutions and Mo substitutions, (e) S substitutions and interstitial sites, and (f) S substitutions and surface adsorptions are stable at Mo-rich and S-rich conditions, respectively. The stable sites depend on the Fermi level (*E*_F_) as the formation energies of dopants linearly depend on their charge states. Here, the ground states at each chemical potential condition refer to the dominant state across the Fermi level. For example, under the Mo-rich conditions, Sc_S_ in (d) is identified as the stable configuration instead of Sc_Mo_.

In more general cases, the stable dopant sites can vary with the chemical potentials and Fermi level. Figure 3 displays the formation energies of the stable sites for the representative dopants from six categories. Figure 3a–c illustrates the dopants with the consistent stable sites regardless of the chemical potential conditions. For example, in Figure 3a, the alkali metal elements Cu and Sn are always thermally stable at the surface site. In the same manner, dopants in Figure 3b,c prefer the Mo-substitution and the S-substitution, respectively, which are irrelevant to the chemical potentials. In contrast, Figure 3d–f displays the dopants that change their preferred sites according to the chemical potentials. For instance, the dopants in Figure 3d prefer S-substitution under the Mo-rich conditions and Mo-substitution under the S-rich condition. In these cases, controlling the chemical environment during the doping processes is crucial to achieve specific dopant configurations and relevant properties. These labels are shown in the periodic table in Figure 1b to indicate the ground states of each dopant under Mo-rich and S-rich conditions. The formation energy diagrams for 27 dopants are also provided in Supporting Information Figures S1 and S2.

After identifying the stable sites for the 27 dopants using the PBEsol functional, their electronic structures and transition levels are explored using the HSE hybrid functionals.^47,48^ Here, we employed two types of Fock exchange mixing parameter (α): (i) the α_KC_ is determined for each donor and acceptor transition to meet the generalized Koopmans’ theorem^42,49−51^ and (ii) the α_gap_ is determined to reproduce the quasiparticle band gap of the ML MoS_2_ (α_gap_ = 0.515) (see the Methods section for details). The α_KC_ has been obtained for 15 donor and 17 acceptor states, as summarized in Figures S3 and S4 and Supporting Information Tables S4 and S5. As shown in Figures S3 and S4, the α_KC_ closely matches α_gap_ = 0.515 for most donor and acceptor transitions, with few exceptions like Au adsorption. Therefore, α_gap_ = 0.515 may be adopted when a single α is required, such as in formation energy calculations.

**Figure 4:**
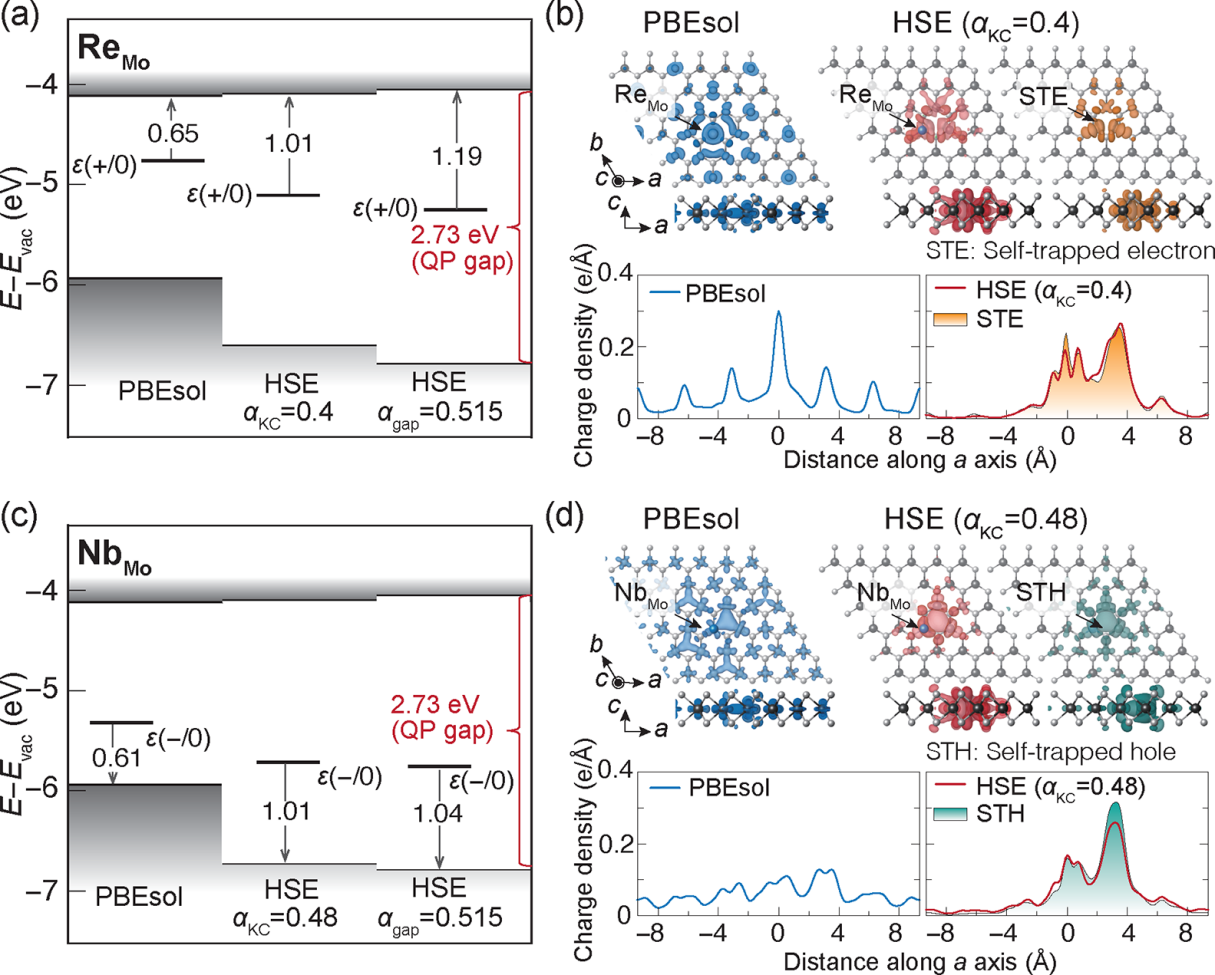
Transition levels with respect to the vacuum level (*E*_vac_) and spatial distribution of the donor and acceptor states introduced by Re_Mo_ and Nb_Mo_, respectively. (a) Donor transition level ε(+/0) of Re_Mo_ and (c) acceptor transition level ε(0/−) of Nb_Mo_ calculated with the PBEsol and HSE functionals. The Fock exchange mixing parameter for the HSE functional is set to α_KC_ = 0.4 for Re_Mo_ and 0.48 for Nb_Mo_ and α_gap_ = 0.515. (b,d) Top panel: partial charge densities of (b) donor state of Re_Mo_ and the self-trapped electron (STE) and (d) acceptor state of Nb_Mo_ and the self-trapped hole (STH) calculated with the PBEsol and HSE functionals at α_KC_. Bottom panel: the planar averages of the partial charge densities along the *a* axis.

Re_Mo_ and Nb_Mo_ are recognized as donors and acceptors for the 2D MoS_2_, with support from both theoretical and experimental studies.^2,25,26,37,38,40,52−54^ Their calculated donor and acceptor transition levels using the PBEsol and HSE hybrid functionals are compared in Figure 4a and c. For the donor state of Re_Mo_ and acceptor state of Nb_Mo_, the α_KC_ values are adjusted to 0.4 and 0.48, respectively, as shown in Figure 4a and c. We highlight significant variations in the donor and acceptor transition levels when comparing the PBEsol and HSE results, whereas the impact of adjusting the α parameter is relatively minor (i.e., not qualitative): the PBEsol functional locates the donor and acceptor transition levels approximately 0.6 eV away from the band edges, while the HSE functionals move these levels to about 1.0 eV. These large differences should stem from a spurious delocalization bias of the PBEsol functional.

As illustrated in Figure 4b and d, while the PBEsol functional predicts a 2D hydrogenic state for both Re_Mo_ and Nb_Mo_,^25,26,55^ the HSE hybrid functionals predict the localized polaronic states bounded to the ionized dopants. It is worth noting that the polaronic states exhibit a spatial distribution similar to that of the self-trapped electron or hole (STE and STH) in the ML MoS_2_, as displayed in Figure 4b and d. Similar trends are also observed in other donor and acceptor states (Figures S5 and S6 in the Supporting Information). The transition levels of the hydrogenic states predicted by PBEsol are located around 0.6 eV, which are much deeper than those of conventional 3D semiconductors. This discrepancy has been attributed to the intrinsic quantum confinement effect caused by the dimensional reduction.^25,26,31,55^ On the other hand, in this study, the HSE hybrid functionals predict the polaronic states, consisting of pairs of a self-trapped carrier and an ionized dopant.

Intriguingly, STE and STH in ML MoS_2_ are found to be energetically unfavorable by 0.21 and 0.42 eV, respectively. However, the electrostatic attractions between an ionized dopant and a self-trapped carrier, summarized in Tables S4 and S5 in the Supporting Information, overcome the formation energies of STE and STH (ranging from −1.0 to −2.0 eV) and strongly stabilize the polaronic states. Displacements of neighboring atoms are also comparable between the self-trapped carriers and polaronic states bound to ionized dopants, as shown in Figure S7 in the Supporting Information. The Kohn–Sham eigenvalues of 15 donors and 17 acceptors, presented in Figures S11 and S12 in the Supporting Information, indicate that the HSE functionals consistently deepen the eigenvalues of the donor and acceptor states.

According to the HSE hybrid functional calculations, deep localized polaronic behaviors are universal for the whole 15 donor and 17 acceptor states. Their partial charge densities, as shown in Figures S12–S19, are strongly localized, including for Re_Mo_ and Nb_Mo_, which are experimentally verified as donor and acceptor dopants, respectively.^2,4,37−40,53,54^ Our results support that the impurity conduction, rather than the band conduction, predominates as the carrier conduction mechanism in 2D MoS_2_, in line with both experimental^4,15,56,58^ and theoretical findings^21,57^ of doped TMDs. This universal polaronic nature suggests that achieving electric conduction in 2D MoS_2_ and similar TMDs may require sufficient doping concentrations, such as a few percent. This is consistent with experiments showing that *n*- and *p*-type doping in MoS_2_ can be achieved through doping with high concentrations of Re and Nb.^4,39,40^

It should be noted that recent experimental results demonstrate small polaron formations in the doped TMDs. Specifically, the scanning tunneling microscopy measurement^59^ of Re_Mo_^0^ dopants in MoS_2_ confirmed the presence of localized in-gap states and local symmetry breaking. It was shown in ref (59) that a DFT calculation of Re_Mo_^0^ using the SCAN + rVV10 functional led to a distorted structure with broken symmetry that is more stable energetically than the symmetric structure (i.e., 2D hydrogenic state). We were able to successfully reproduce their results with our calculation setting using the same SCAN + rVV10 functional, which shows that the electronic and atomic structures are the same as the small polaron states we found (see Figure S20). Additionally, photoluminescence measurements of redoped TMDs^60,61^ also exhibited impurity-originated signals attributed to the split-off defect bands, which can originate from the localized polaronic state.

Since the polaronic states are strongly tied around the dopants, they are expected to be less sensitive to the substrate and the number of stacking layers of MoS_2_. In fact, in the PBEsol calculations, the hydrogenic state in 3D MoS_2_ becomes further spatially delocalized in the out-of-plane direction, reducing the donor transition level from 0.67 to 0.24 eV, as shown in Figure S8 in the Supporting Information. This result is consistent with previous reports.^25,26^ In contrast, the localized polaronic behavior in the Koopmans-compliant hybrid functional calculation is shown to be confined in a single layer of 3D MoS_2_ despite the shallower transition level with a smaller Koopmans-compliant (α_KC_) parameter compared to 2D MoS_2_.

As demonstrated in the case of Re_Mo_ and Nb_Mo_ in Figure 4, using the HSE functional changes the donor/acceptor states even *qualitatively*, resulting in significant shifts of transition levels toward the midgap. The transition levels of the Li and Au adsorptions are shown in Figure S9 in the Supporting Information. They are representative examples of the hydrogenic donor states and deep donor states, respectively, predicted by the PBEsol functional. The position of the deep donor state caused by Au adsorption is almost constant with respect to the vacuum level when changing the exchange–correlation functional. On the other hand, the position of the Li adsorption exhibits a large shift toward the midgap when adopting the HSE functional: the HSE functional predicts formation energies for Li adsorption at *q* = 0 to be significantly lower than the PBEsol functional. This energy lowering is attributed to the small polaron formation, which is consistent with the defect formation energies of several Mo substitutions (Tc_Mo_, Re_Mo_, V_Mo_, Nb_Mo_, and Ta_Mo_) and surface adsorptions of alkali metals (Li, Na, and K) (see Supporting Information Tables S2 and S3). This suggests that adjusting the band edge positions does not effectively correct the transition levels of such cases, and reducing self-interaction error by using hybrid functionals is crucial for analyzing localized donors and acceptors in MoS_2_.

**Figure 5:**
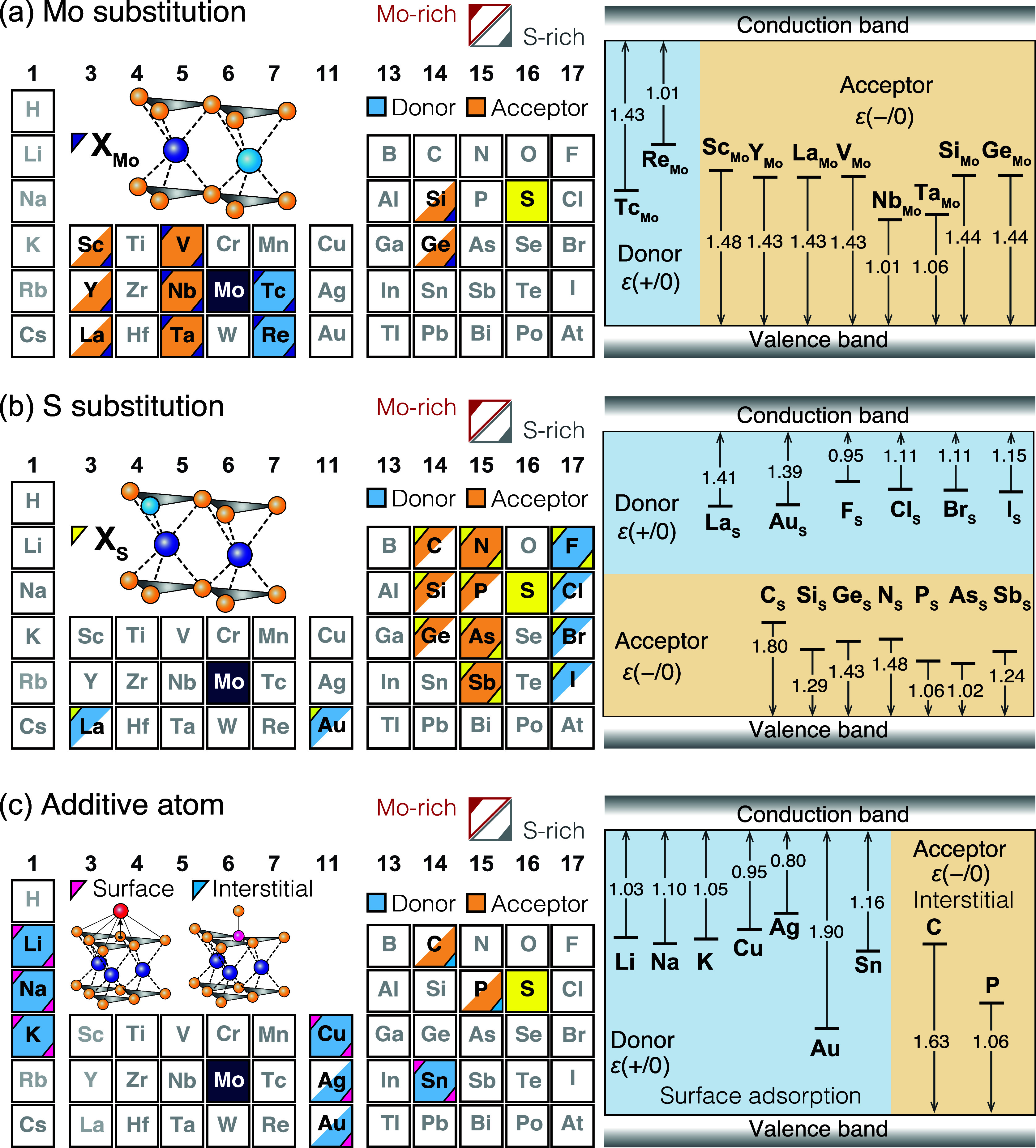
Ground states of the 27 dopants achieved with chemical potentials at the Mo-rich to S-rich conditions. The notations in the periodic tables are the same as those in Figure 1b, but (a) Mo substitutions, (b) S substitutions, and (c) surface adsorptions and lattice interstitials are distinguished. In the right panels, the donor and acceptor transition levels obtained with the Koopmans-compliant HSE hybrid functionals are shown in eV.

Finally, we discuss the transition levels of the ground states obtained by using the HSE hybrid functionals at α_KC_. Our calculations predict 10 types of Mo substitutions, 13 types of S substitutions, and 9 types of additive atoms (7 surface adsorptions and 2 interstitials) to be ground states under the Mo-rich and/or S-rich conditions. As shown in Figure 5a, early transition metals (Sc, Y, and La), groups 5 and 7 elements adjacent to Mo on the periodic table (V, Nb, Ta, Tc, and Re), and group 14 elements (Si and Ge) preferentially substitute the Mo site. Among them, elements in groups 5 and 7 exhibit acceptor and donor characteristics, respectively, due to having one fewer and additional valence electron compared to Mo, respectively. The other Mo-substituted elements mostly exhibit donor states, where localized in-gap states are found even in the PBEsol calculations. Elements adjacent to S on the periodic table prefer the S substitutions (Figure 5b). A few exceptions also exist: the S substitutions with Au and La are stable under the Mo-rich conditions and behave as donors. Figure 5c presents the group of surface adsorptions and lattice interstitials. It is found that cationic elements are stable as surface adsorptions, whereas C and P are thermally stabilized as lattice interstitials. Because of their cationic and anionic characteristics, surface adsorptions and lattice interstitials serve as donors and acceptors, respectively. The surface adsorption of alkali metals (Li, Na, and K) induces small electron polarons when adopting the hybrid functionals (α = α_KC_). For other surface adsorptions (Cu, Ag, Au, and Sn), the localized occupied in-gap states are found even with the semilocal PBEsol functional. The interstitial C and P atoms occupy the S site and displace the S atom on the C and P interstitials. The structure and acceptor transition levels are similar to those of As at the I2 site.

## Conclusions

In summary, we provided general insight into the elemental doping of 2D MoS_2_. The stability and electronic structures of 27 dopants residing in atomic substitutions, surface adsorptions, and lattice interstitials were systematically explored. This study offers a comprehensive perspective on elemental doping of ML MoS_2_ under different chemical conditions, as summarized in Figure 1b. To address the limitations of semilocal functionals, the Koopmans-compliant hybrid functionals have been systematically applied to explore stable sites of the dopants. Unlike the PBEsol functional, the hybrid functionals provide a *universal* polaronic behavior of donors and acceptors in ML MoS_2_ with deep transition levels 1.0 eV away from the band edges. This polaronic description includes the Re and Nb dopants, which are experimentally known *n*-type and *p*-type dopants, respectively, and persists even in dielectric medium such as bulk MoS_2_. This universal polaron formation of a wide range of dopants suggests that impurity conduction may serve as the predominant mechanism for the electric conduction of MoS_2_. This work broadens insight into elemental doping in MoS_2_ and other TMDs, laying the foundation for refined elemental doping strategies in 2D TMDs.

## Methods

### DFT Calculations

We calculated the electronic structures of the pristine and defective MoS_2_ based on the DFT with the projector-augmented wave (PAW) approach^62^ as implemented in the Vienna Ab initio Simulation Package (VASP).^63−66^ The cutoff energy of plane waves was set to 270 eV for most calculations, while the 400 eV cutoff energy was selectively employed when atoms with small PAW radii (N, C, O, and F) were included in the calculations. Γ-centered 12 × 12 × 1 and 12 × 12 × 3 Monkhorst–Pack *k*-point grids^67^ were adopted for the monolayer and bulk 2H–MoS_2_ in their unit cells.

Point defect calculations were performed with 6 × 6 × 1 supercells of the monolayer MoS_2_, with sampling of the Γ-point in the reciprocal space. Input files for unit cell and supercell calculations for the VASP package were generated with the VISE package (ver. 0.8.1).^68^ The PBEsol functional^46^ was employed for the calculations of chemical potentials and stability of dopants, while the HSE hybrid functional^47,48^ was employed for calculating the prescreened stable configurations using PBEsol. The screening distance in the reciprocal space for the HSE hybrid functional was fixed to μ = 0.208 Å^–1^. The Fock exchange mixing parameter α = 0.515 reproduces the experimental and theoretical quasiparticle gap of the monolayer MoS_2_ (∼2.73 eV) and it is referred to as α_gap_ in the main text.^31,34,69^ The relaxed lattice constants of the monolayer MoS_2_ obtained with the PBEsol and HSE (α_gap_) are *a*_PBEsol_ = 3.14 Å and *a*_hse*-*gap_ = 3.13 Å, respectively. The parameter α is also tuned to satisfy the generalized Koopman’s condition, which is referred to as α_KC_. The α_KC_ ranges from 0.289 to 0.817 depending on the donor and acceptor states (see the text for details). We used a fixed lattice constant of *a*_hse*-*gap_ = 3.13 Å. Within the α_KC_ range, the variation in the in-plane lattice constant is less than 1%.

The dielectric tensors of the monolayer and bulk MoS_2_ were computed through the density-functional perturbation theory^70,71^ and self-consistent response to finite electric fields^72−74^ using the PBEsol and HSE hybrid functionals, respectively. Pristine structures of MoS_2_ were optimized until the Hellman–Feynman forces exerting on atoms became less than 0.005 eV/Å, and MoS_2_ supercells with defects were relaxed with fixed lattice parameters until atomic forces became less than 0.03 eV/Å. For the structural optimization of the bulk MoS_2_, the DFT-D3 method of Grimme with zero-damping function was applied.^75^

### Defect Formation Energy

Defect formation energy (Δ*E*_f_) is defined as
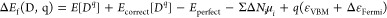
where *E*[*D*^*q*^] is the total energy of the defective supercell, including the defect *D* in the charge state *q* (*D*^*q*^), and *E*_correct_[*D*^*q*^] is the correction energy for the charged defect *D*^*q*^ under the periodic boundary condition, *N*_*i*_ is the number of added or removed atoms, μ_*i*_ is the chemical potential of the element *i*, and ε_VBM_ and Δε_Fermi_ are the eigenvalues of the valence band maximum (VBM) and the relative Fermi level referenced to the ε_VBM_, respectively.^76−78^ The Mo and S substations, surface adsorptions (on the three site candidates), and lattice interstitials (at two interlattice sites) of 27 dopants were considered in the calculations (see the main text for details). We calculated the chemical potentials of μ_Mo_, μ_S_, and μ_*X*_, where *X* means the dopant element from the total energies of the competing phases, which were retrieved from the Material Project Database.^79^ The pydefect code^68^ was utilized to construct point defect structures and chemical potential diagrams between Mo, S, and 27 different types of dopants and plot the defect formation energy diagrams. The chemical potentials of μ_Mo_, μ_S_, and μ_X_ in Mo-rich and S-rich conditions are listed in Table S1 in the Supporting Information.

### Corrections on Formation Energies of Charged Point Defects

The *E*_correct_[*D*^*q*^] term is computed with the pydefect_2D package,^28^ employing the correction scheme for charged point defects in two-dimensional systems proposed by Noh et al., Komsa et al., Sundararaman and Ping, and Kumagai.^24,27,28,80^ For evaluating *E*_correct_[*D*^*q*^] terms for the charged defects in the monolayer MoS_2_, the dielectric profile is approximated with a step function with parameters *s* = 0.5 Å, *w* = 7.15 Å, and *w*_z_ = 7.15 Å, where s is the edge smearing parameter, and *w* and *w*_z_ are the widths of the step function for in-plane and out-of-plane dielectric constants, respectively. Details of the correction are found in ref (28). We note that a conventional potential alignment correction is unnecessary when the image-charge correction is properly applied to the defect formation energy.^81^ The computed *E*_correct_[*D*^*q*^] terms were parsed into the pydefect package for evaluating defect formation energies and charge transition levels.

### Charge Transition Level

The thermodynamic charge transition level is defined as the point of the Fermi level at which the most energetically stable charge state of a defect changes. From the definition of the defect formation energy, the transition level ε(*q*/*q*’) is evaluated as ε(*q*/*q*’) = [Δ*E*_f_(*D*,*q*’)−Δ*E*_f_(*D*,*q*)]/(*q* – *q*’), where Δ*E*_f_(*D*,*q*) = Δ*E*_f_(*D*,*q*’) at Δε_Fermi_ = ε(*q*/*q*’). When *q* > *q*’, the stable charge state is *q* if the Fermi level is lower than ε(*q*/*q*′), and vice versa. The ε(0/−) and ε(0/+) values measured from the VBM and the conduction band minimum correspond to the single acceptor and donor levels, respectively.

### Generalized Koopmans’ Condition and Non-Koopmans Energy

For polaronic occupied donor and unoccupied acceptor states, the generalized Koopmans’ conditions, which is satisfied for self-interaction-free DFT calculations, are given as Δ*E* = *E*(*q* = 0) – *E*(*q* = +1) = ε_*i*_ for donor states and Δ*E* = *E*(*q* = −1) – *E*(*q* = 0) = ε_*i*_ for acceptor states, where ε_i_ is the eigenvalue of the polaronic donor and acceptor state.^42,82,83^ The non-Koopmans energy Δ*E*_NK_ = ε_*i*_ – Δ*E* is calculated for each localized donor and acceptor states to measure the degree of accordance of the generalized Koopmans’ condition. The Δ*E*_NK_ values estimated using α_gap_ = 0.515 are tabulated in Tables S2 and S3. The α_KC_ values are determined for individual polaronic in-gap states with achieving |Δ*E*_NK_| less than 0.05 eV.

### Binding Energy of the Ionized Dopant and Self-Trapped Carrier

The binding energy between an ionized donor and the self-trapped electron is calculated as *E*_b,donor_ = [*E*(*D*, *q* = 0) + *E*_perfect_] – [*E*(*D*, *q* = +1) + *E*_STE_], where *E*(*D*, *q* = 0) and *E*(*D*, *q* = +1) are the total energies of the dopant *D* of the charge state *q* = 0 and *q* = +1, *E*_perfect_ is the total energy of the perfect supercell, and *E*_STE_ is the total energy of the supercell including the self-trapped electron. In analogy to *E*_b,donor_, the binding energy between an ionized acceptor and the self-trapped hole is calculated as *E*_b,acceptor_ = [*E*(*D*, *q* = 0) + *E*_perfect_] – [*E*(*D*, *q* = −1) + *E*_STH_], where *E*_STH_ is the total energy of the supercell including the self-trapped hole. The calculated binding energies *E*_b,donor_ and *E*_b,acceptor_ for 15 donors and 17 acceptors are listed in Table S4 and Table S5 in the Supporting Information.

## Supporting Information Available

Information of 27 dopants in MoS_2_ including formation energies, donor and acceptor transitions, shallow-to-deep transition, polaronic lattice distortion, dielectric screening effect, band-edge shift effect, eigenvalue spectra, and partial charge densities of donors and acceptors; chemical potentials at Mo-rich and S-rich condition; defect formation energy of donor and acceptor dopants; and Koopmans-compliant mixing parameter of the HSE functional and carrier–dopant binding energy of polaronic donors (PDF)
