# Supplementary material for: Universal Polaronic Behavior in Elemental Doping of MoS2 from First-Principles
Source: ACS Nano. 2024 Dec 2;18(50):33988–97. doi: 10.1021/acsnano.4c08366 (PMC11656834; doi:10.1021/acsnano.4c08366)
Supplement: Supplementary file 1 — nn4c08366_si_001.pdf [file nn4c08366_si_001.pdf]

Supporting Information:

Universal polaronic behavior in elemental doping of MoS_2 from first principles

Soungmin Bae[a*], Ibuki Miyamoto[a], Shin Kiyohara[a], and Yu Kumagai[a*]

[a]Institute for Materials Research, Tohoku University, 2-1-1 Katahira, Aoba-ku, Sendai, 980-8577, Japan

E-mail: bae.soungmin.d6@tohoku.ac.jp; yukumagai@tohoku.ac.jp

Contents

Figure S1. Formation energy of dopants for stable configurations in the monolayer MoS_2. ----------------- 1

Figure S2. Formation energy of dopants for stable configurations in the monolayer MoS_2. ----------------- 2

Figure S3. Donor transition levels [$\varepsilon(+/0)$] levels of donor dopants of the monolayer MoS_2 depending on the Fock exchange mixing parameter (α) of the HSE hybrid functional. --------------------------------------- 3

Figure S4. Acceptor transition levels [$\varepsilon(+/0)$] levels of donor dopants of the monolayer MoS_2 depending on the Fock exchange mixing parameter (α) of the HSE hybrid functional.--- 4

Figure S5. Shallow-to-deep transition of perturbed host states (PHS) into polaronic deep donor states induced by eliminating self-interaction of the PBEsol functional by adopting the HSE functional. ---------- 5

Figure S6. Shallow-to-deep transition of perturbed host states (PHS) into polaronic deep acceptor states induced by eliminating self-interaction of the PBEsol functional by adopting the HSE functional. ---------- 6

Figure S7. Lattice distortion induced by the small polaron formation of the localized self-trapped electron and self-trapped hole in the monolayer MoS_2 obtained with the Koopmans-compliant HSE functional. --- 7

Figure S8. Dielectric screening effect on the donor transition level and spatial distribution of the donor state of Re_{Mo} in the monolayer and bulk MoS_2. -- 8

Figure S9. Formation energies, transition levels, eigenvalue spectra, and partial charge densities of donor states of surface adsorptions of Li and Au atom on the monolayer MoS_2. -------------------------------------- 9

Figure S10. Eigenvalue spectra of 15 types of donors including Mo substitutions (Tc_{Mo}, and Re_{Mo}), S substitutions (La_S, Au_S, Cl_S, F_S, Br_S, and I_S) and surface adsorptions (Li, Na, K, Cu, Ag, Au, and Sn) in the monolayer MoS_2. -- 10

Figure S11. Eigenvalue spectra of 17 types of acceptors including Mo substitutions (Sc_{Mo}, Y_{Mo}, La_{Mo}, V_{Mo}, Si_{Mo}, Ta_{Mo}, Nb_{Mo}, and Ge_{Mo}), lattice interstitials (C, and P), and S substitutions (C_S, Si_S, Ge_S, N_S, P_S, As_S, and Sb_S) in the monolayer MoS_2. -- 11

Figure S12. Eigenvalue spectra and partial charge densities of polaronic donor states. --------------------- 12

Figure S13. Eigenvalue spectra and partial charge densities of polaronic donor states. --------------------- 13

Figure S14. Plane averaged partial charge densities of the self-trapped electron (STE) and the polaronic donor states in the monolayer MoS_2. --- 14

Figure S15. Plane averaged partial charge densities of the self-trapped electron (STE) and the polaronic donor states in the monolayer MoS_2. --- 15

Figure S16-17. Eigenvalue spectra and partial charge densities of polaronic acceptor states. -------------- 16

Figure S18-19. Plane averaged partial charge densities of the self-trapped hole (STH) and the polaronic acceptor states in the monolayer MoS_2. --- 18

Figure S20. Comparison of Re_{Mo} (q=0) results obtained with SCAN+rVV10 and HSE functionals ------- 20

Table S1. Chemical potentials at Mo-rich and S-rich condition. --- 21

Table S2. Defect formation energy of donor dopants at the Mo-rich and S-rich conditions and their difference between the PBEsol and HSE results. --- 22

Table S3. Defect formation energy of acceptor dopants at the Mo-rich and S-rich conditions and their difference between the PBEsol and HSE results. --- 22

Table S4. Koopmans-compliant mixing parameter (α_{KC}), the transition levels [ε(+/0)] of donor dopants obtained with the PBEsol functional and HSE functionals with Fock exchange mixing parameters of α_{gap}= 0.515 and α_{KC}, and carrier–dopant binding energy of polaronic donors ($E_{b,donor}$). ------------------------------ 23

Table S5. Koopmans-compliant mixing parameter (α_{KC}), the transition levels [ε(+/0)] of acceptor dopants obtained with the PBEsol functional and HSE functionals with Fock exchange mixing parameters of α_{gap}= 0.515 and α_{KC}, and carrier–dopant binding energy of polaronic acceptors ($E_{b,acceptor}$). ------------------------ 23

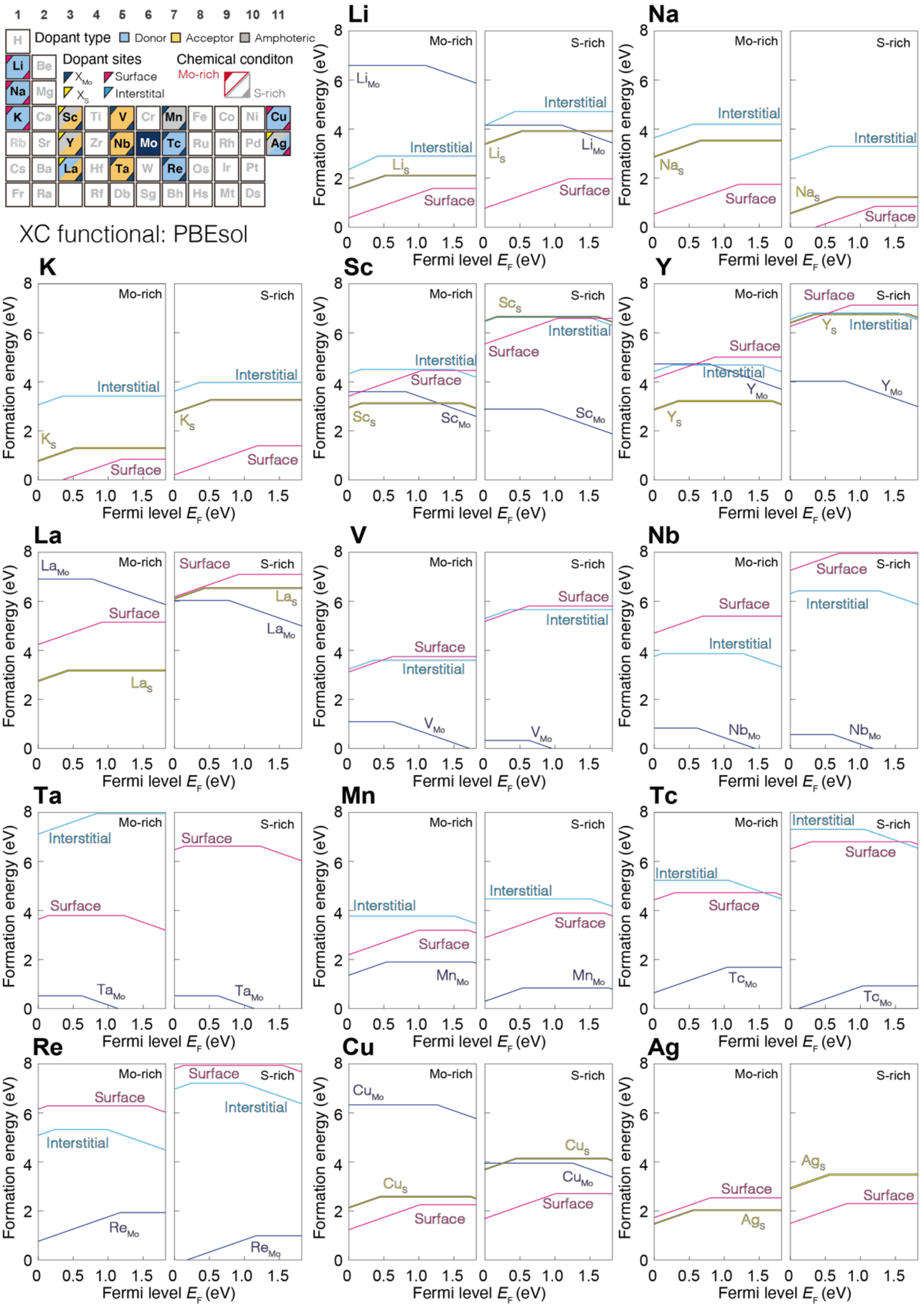

Figure S1. Formation energy of dopants for stable configurations in the monolayer MoS_2. Formation energy of stable sites belongs to substitutional (X_{Mo} and X_S), surface adsorption, and interstitial sites under

Mo-rich and S-rich conditions of 14 dopants calculated with the PBEsol functional. The most stable configurations of each dopant at Mo-rich and S-rich conditions are labeled in the periodic table.

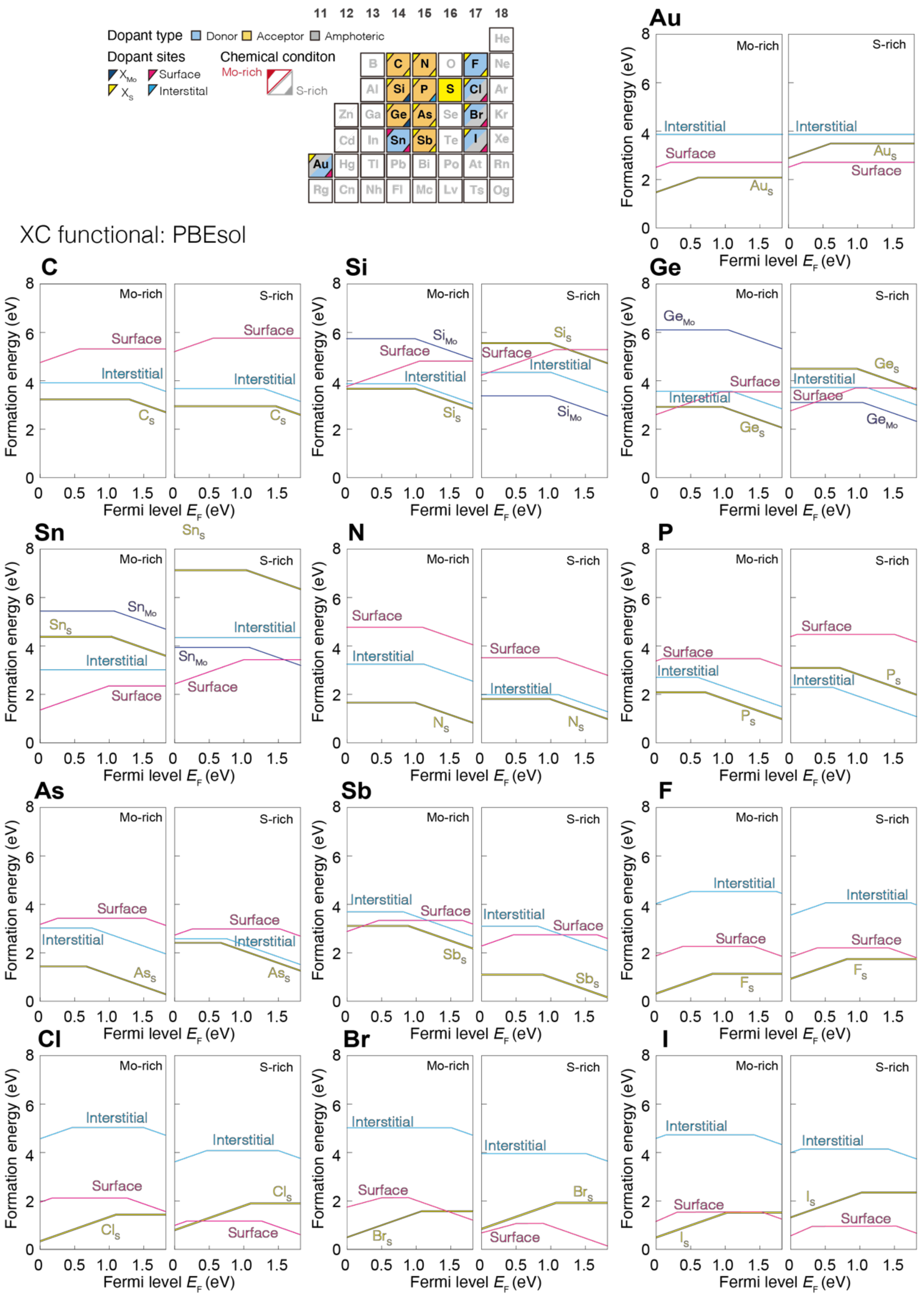

Figure S2. Formation energy of dopants for stable configurations in the monolayer MoS_2. Formation energy of stable sites belongs to substitutional (X_{Mo} and X_S), surface adsorption, and interstitial sites under

Mo-rich and S-rich conditions of 14 dopants calculated with the PBEsol functional. The most stable configurations of each dopant at Mo-rich and S-rich conditions are labeled in the periodic table.

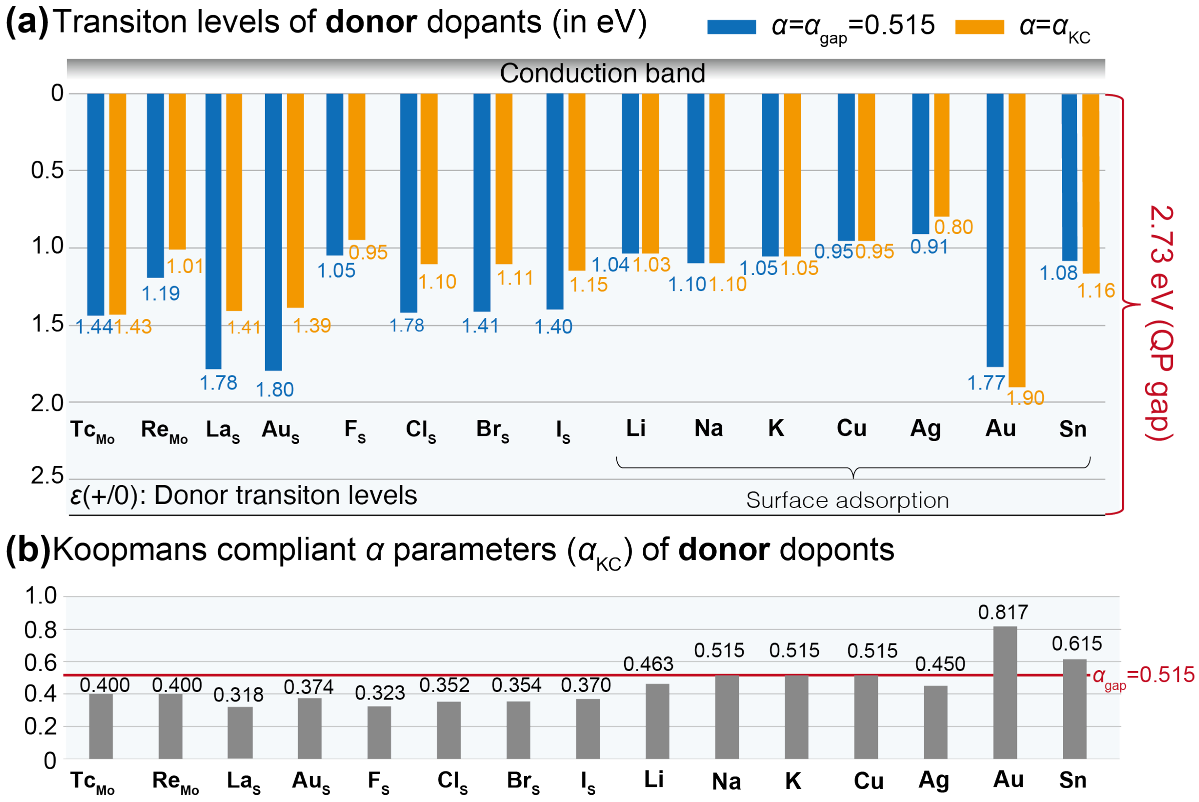

Figure S3. Donor transition levels [$\varepsilon(+/0)$] levels of donor dopants of the monolayer MoS_2 depending on the Fock exchange mixing parameter (α) of the HSE hybrid functional. (a) Comparison of donor transition levels obtained using the HSE hybrid functional (the screening parameter is set to μ = 0.208 Å^{-1}) with mixing parameters of α_{gap} = 0.515 and α_{KC} that reproduces the quasiparticle gap of monolayer MoS_2 and satisfies Koopman's condition for each dopant. (b) The list of α_{KC} parameters fulfilling the Koopman's condition of each donor state i.e., the occupied in-gap state involved in the donor transition.

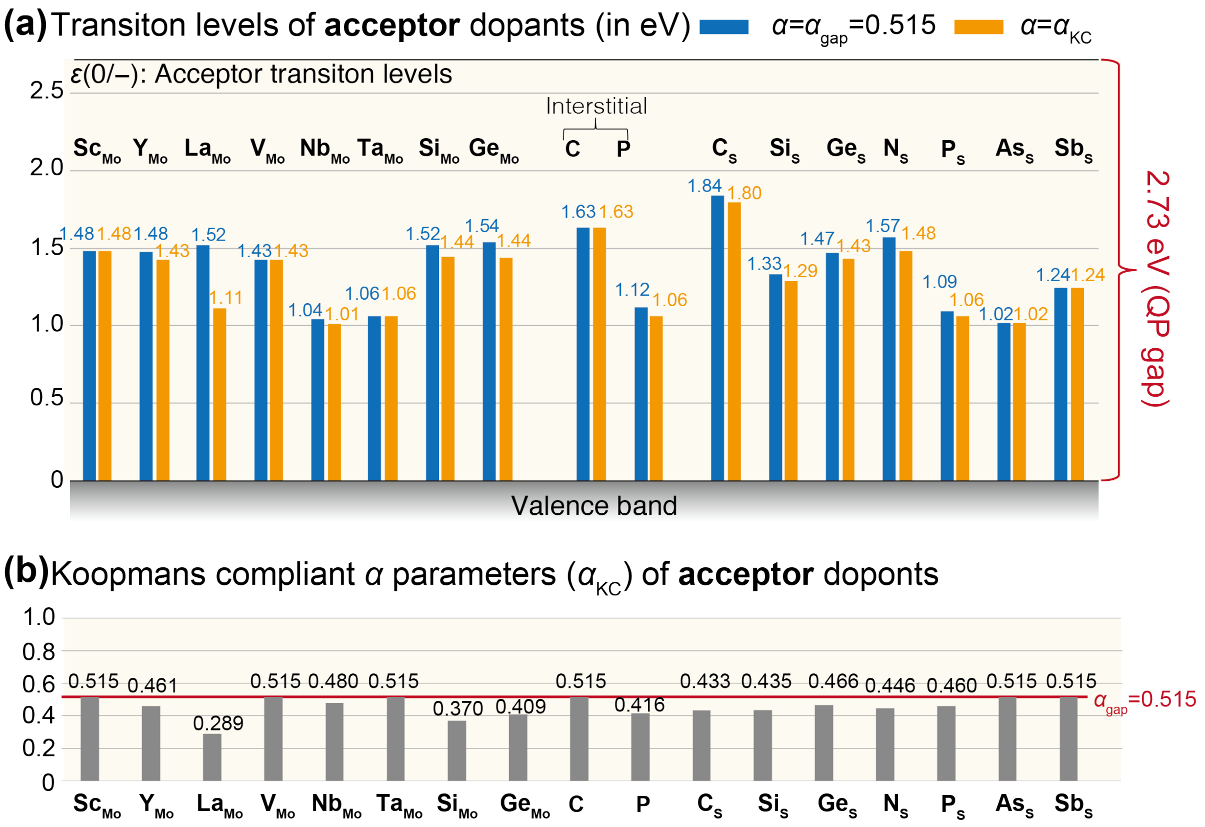

Figure S4. Acceptor transition levels [$\varepsilon(+/0)$] levels of donor dopants of the monolayer MoS_2 depending on the Fock exchange mixing parameter (α) of the HSE hybrid functional. (a) Comparison of donor transition levels obtained using the HSE hybrid functional (the screening parameter is set to μ = 0.208 $Å^{-1}$) with mixing parameters of α_{gap} = 0.515 and α_{KC} that reproduces the quasiparticle gap of monolayer MoS_2 and satisfies Koopman's condition for each dopant. (b) The list of α_{KC} parameters fulfilling the Koopman's condition of each donor state i.e., the unoccupied in-gap state involved in the acceptor transition.

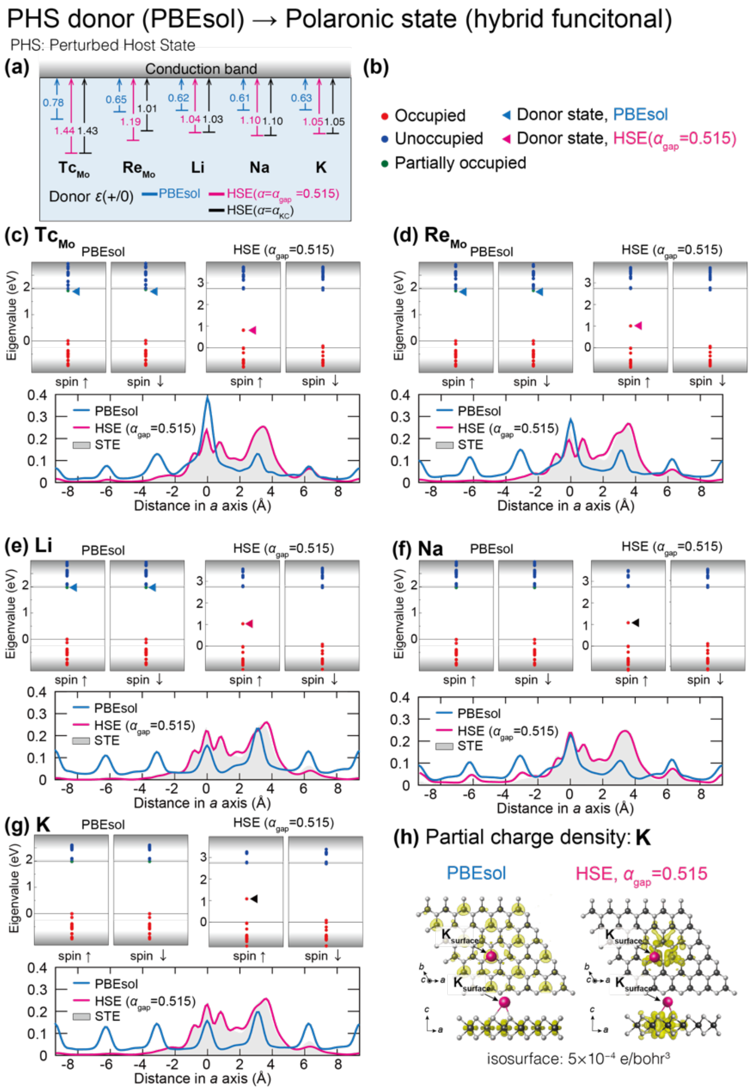

Figure S5. Shallow-to-deep transition of perturbed host states (PHS) into polaronic deep donor states induced by eliminating self-interaction of the PBEsol functional by adopting the HSE functional. (a) Donor transition levels of Mo substitutions (Tc_{Mo} and Re_{Mo}) and surface adsorptions of alkali metals (Li, Na, and K) obtained with the PBEsol and HSE hybrid functionals (the screening parameter μ= 0.208 Å^{-1}) of the Fock exchange mixing parameters α= 0.515 (=α_{gap}) and α_{KC}. (b) The labels presented in eigenvalue spectra denote the electron occupation of states (occupied, unoccupied, and partially occupied) at the Γ point in and the donor states of the PBEsol and HSE functionals (α_{gap} = 0.515). (c-g) The eigenvalue spectrum of donor dopants (Tc_{Mo}, Re_{Mo}, Li, Na, and K) and the plane averaged partial charge density of the donor states are marked in the eigenvalue spectra compared with that of the self-trapped electron (STE). While the donor states of the PBEsol functional are widely delocalized over the cell (i.e., the perturbed host state, PHS), the HSE functional derives a *shallow-to-deep* transition by localizing the donor states into the self-trapped electron, as shown in the similarity of the plane averaged partial charge density of the donor states and the self-trapped electron state. (h) The partial charge density of the donor state of the surface adsorption of potassium (K) obtained with the PBEsol functional and HSE functional, which give the *delocalized* PHS state and the *localized* polaronic state, respectively.

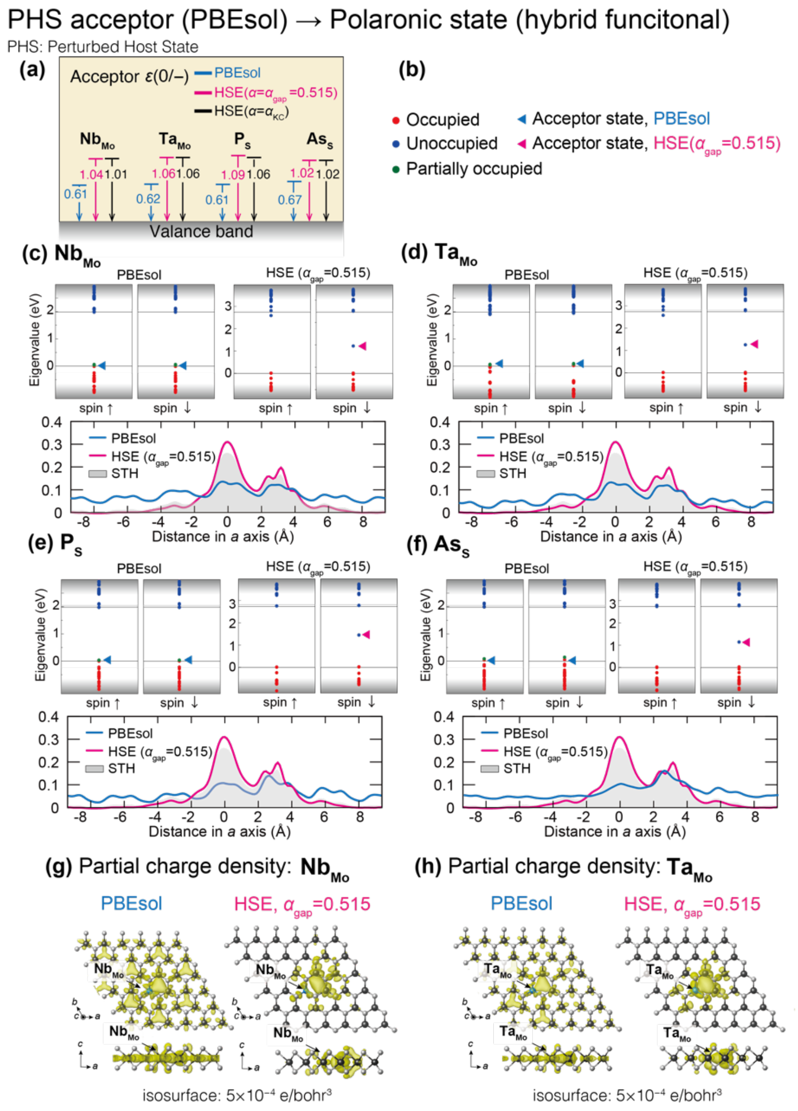

Figure S6. Shallow-to-deep transition of perturbed host states (PHS) into polaronic deep acceptor states induced by eliminating self-interaction of the PBEsol functional by adopting the HSE functional. (a) Acceptor transition levels of Mo substitutions (Nb_{Mo} and Ta_{Mo}) and S substitutions (P_S and As_S) obtained with the PBEsol and HSE hybrid functionals (the screening parameter μ= 0.208 Å^{-1}) of the Fock exchange mixing parameters α= 0.515 (=α_{gap}) and α_{KC}. (b) The labels presented in eigenvalue spectra denote the electron occupation of states (occupied, unoccupied, and partially occupied) at the Γ point and the donor states of the PBEsol and HSE functionals (α_{gap} = 0.515). (c-g) The eigenvalue spectrum of donor dopants (Nb_{Mo}, Ta_{Mo}, P_S, and As_S) and the plane averaged partial charge density of the donor states are marked in the eigenvalue spectra compared with that of the self-trapped hole (STH). While the acceptor states of the PBEsol functional are widely delocalized over the cell (i.e., the perturbed host state, PHS), the HSE functional derives a *shallow-to-deep* transition by localizing the acceptor states into the self-trapped hole, as shown in the similarity of the plane averaged partial charge density of the acceptor states and the self-trapped hole state. (h) The partial charge density of the donor state of Nb_{Mo}, Ta_{Mo} obtained with the PBEsol functional and HSE functional, which give the *delocalized* PHS state and the *localized* polaronic state, respectively.

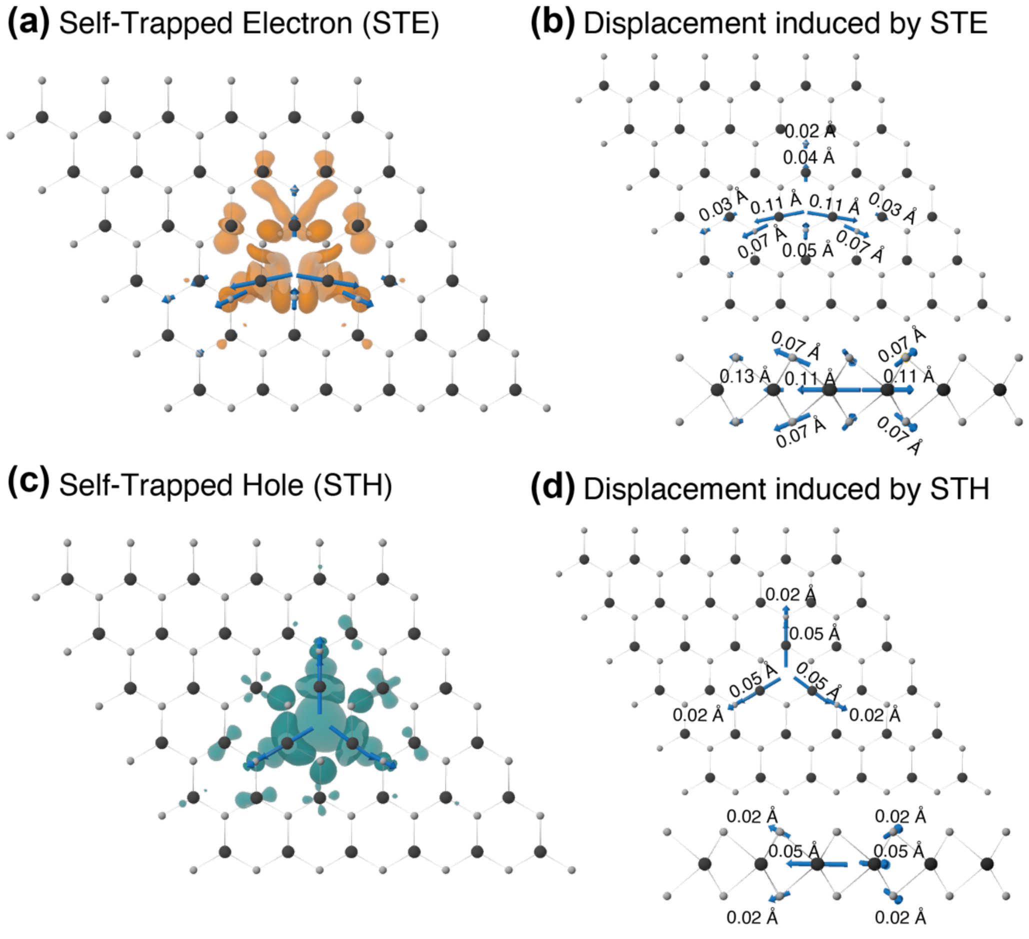

Figure S7. Lattice distortion induced by the small polaron formation of the localized self-trapped electron and self-trapped hole in the monolayer MoS_2 obtained with the Koopmans-compliant HSE functional. (a) The localized charge distribution of the self-trapped electron and the induced relative atomic displacements of Mo (black) and S (gray) atoms, which form a small electron polaron. (b) The atomic displacements induce by the self-trapped electron formation. (c) The localized charge distribution of the self-trapped hole and the induced relative atomic displacements of Mo and S atoms, which form a small hole polaron. (d) The atomic displacements induce by the self-trapped hole formation.

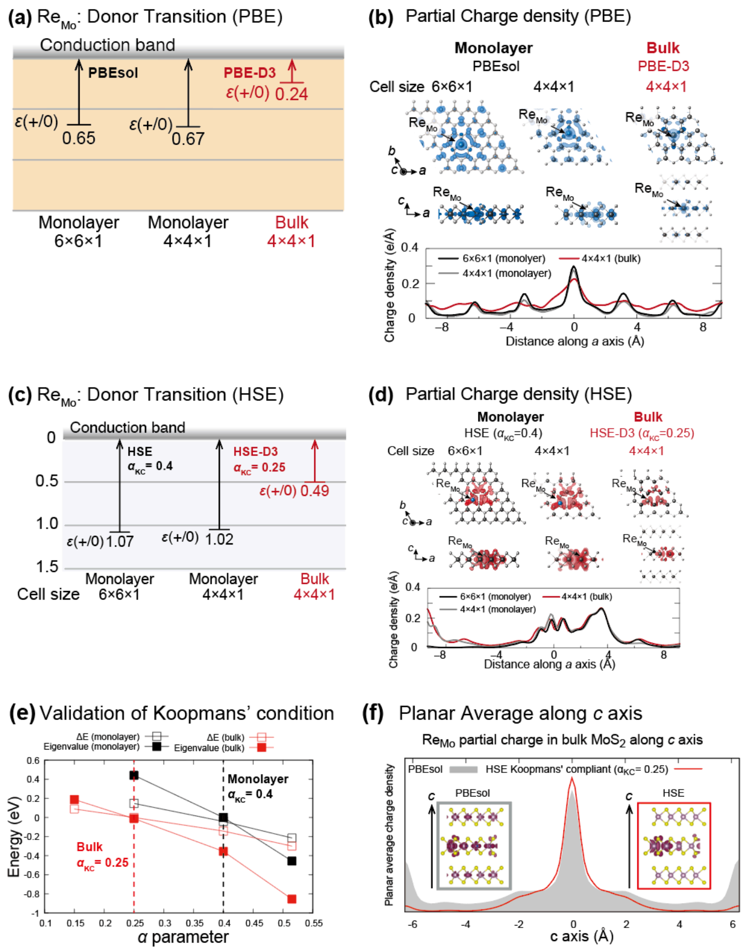

Figure S8. Dielectric screening effect on the donor transition level and spatial distribution of the donor state of Re$_{Mo}$ in the monolayer and bulk MoS_2. (a) The donor transition level ε(+/0) of Re$_{Mo}$ calculated with the semi-local PBE functionals (PBEsol and PBE-D3) at α_{KC}, where Re$_{Mo}$ is in the monolayer (cell size: 6×6×1 and 4×4×1) and bulk (3D) MoS_2 (cell size: 4×4×1). (b) Partial charge densities of the Re$_{Mo}$ donor state in monolayer and bulk MoS_2 obtained with PBEsol and their plane averaged plots along the *a* axis. (c) The donor transition level ε(+/0) of Re$_{Mo}$ calculated with the Koopmans compliant HSE functionals, where Re$_{Mo}$ is in the monolayer (cell size: 6×6×1 and 4×4×1) and bulk MoS_2 (cell size: 4×4×1). (d) Partial charge densities of the Re$_{Mo}$ donor state in monolayer and bulk MoS_2 obtained with the Koopmans compliant HSE functionals and their plane averaged plots along the *a* axis. (e) The *α* parameter dependence of the eigenvalue of the donor state (q=0) and total energy difference $\Delta E = E(q=0) - E(q=+1)$ of Re$_{Mo}$ in monolayer and bulk MoS_2. This determines the Koopmans compliant *α* parameters of the Re$_{Mo}$ donor state as α_{KC} = 0.4 and 0.25 for the monolayer and bulk MoS_2, respectively. (f) The planar averaged partial charge of the Re$_{Mo}$ donor state in bulk MoS_2 obtained with PBE-D3 and HSE (α_{KC} = 0.25) functionals along the *c* axis (the stacking direction).

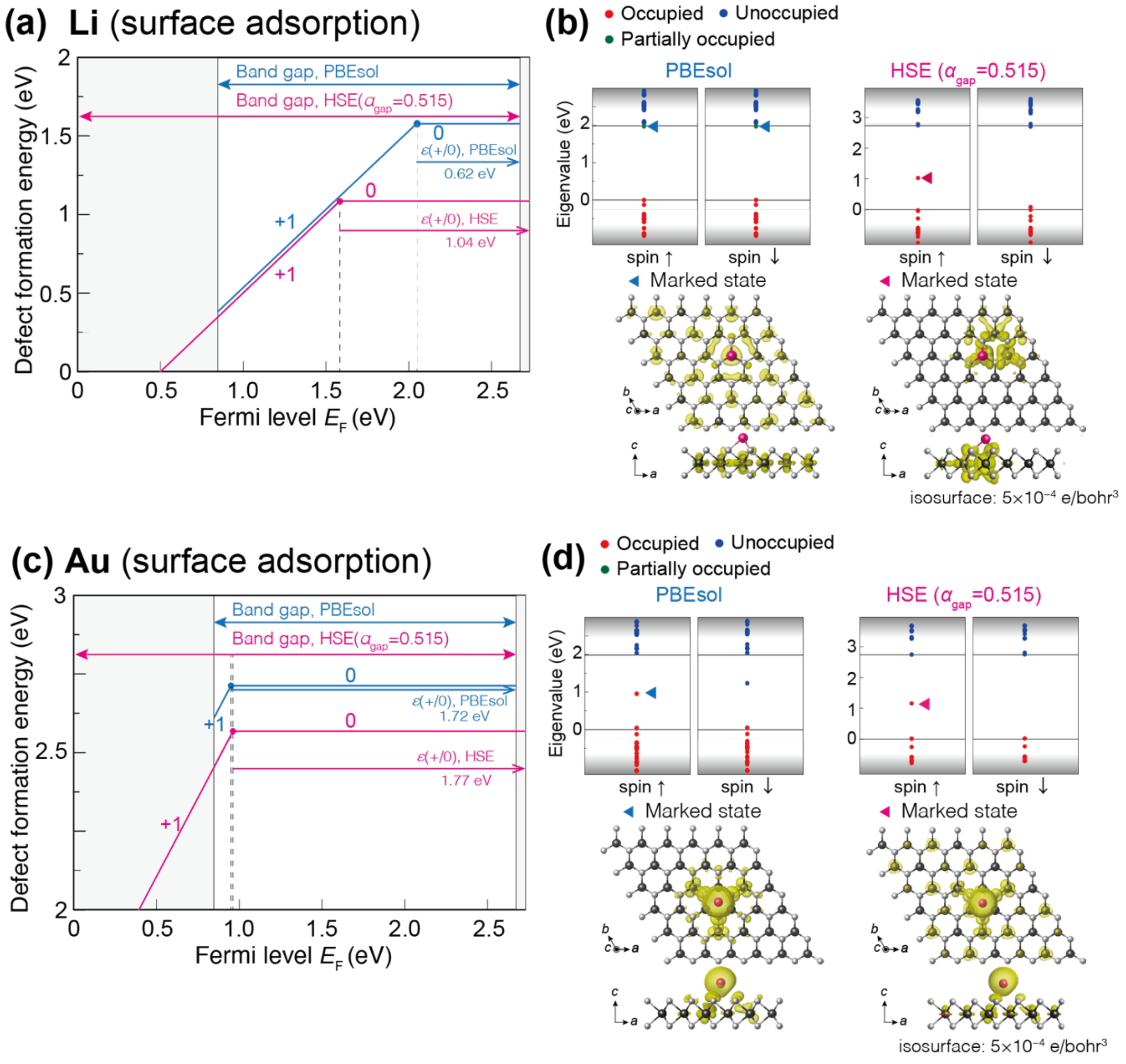

Figure S9. Formation energies, transition levels, eigenvalue spectra, and partial charge densities of donor states of surface adsorptions of Li and Au atom on the monolayer MoS_2. (a) Formation energy of the surface adsorption of Li atom calculated with the PBEsol and HSE functional ($\alpha = \alpha_{gap} = 0.515$). The band edge positions of the monolayer MoS_2 obtained with the PBEsol functional is aligned to the band edges of the HSE functional where the valance band maximum (VBM) of the monolayer MoS_2 obtained with the HSE functional is set to 0. (b) The eigenvalue spectra of the surface adsorption of Li atom and the partial charge densities of the donor state (marked with the triangle symbol) obtained with the PBEsol and HSE functional. (c) Formation energy of the surface adsorption of Au atom calculated with the PBEsol and HSE functional ($\alpha = \alpha_{gap} = 0.515$). The band edge positions of the monolayer MoS_2 obtained with the PBEsol functional is aligned to the band edges of the HSE functional where the valance band maximum (VBM) of the monolayer MoS_2 obtained with the HSE functional is set to 0. (b) The eigenvalue spectra of the surface adsorption of Au atom and the partial charge densities of the donor state (marked with the triangle symbol) obtained with the PBEsol and HSE functional.

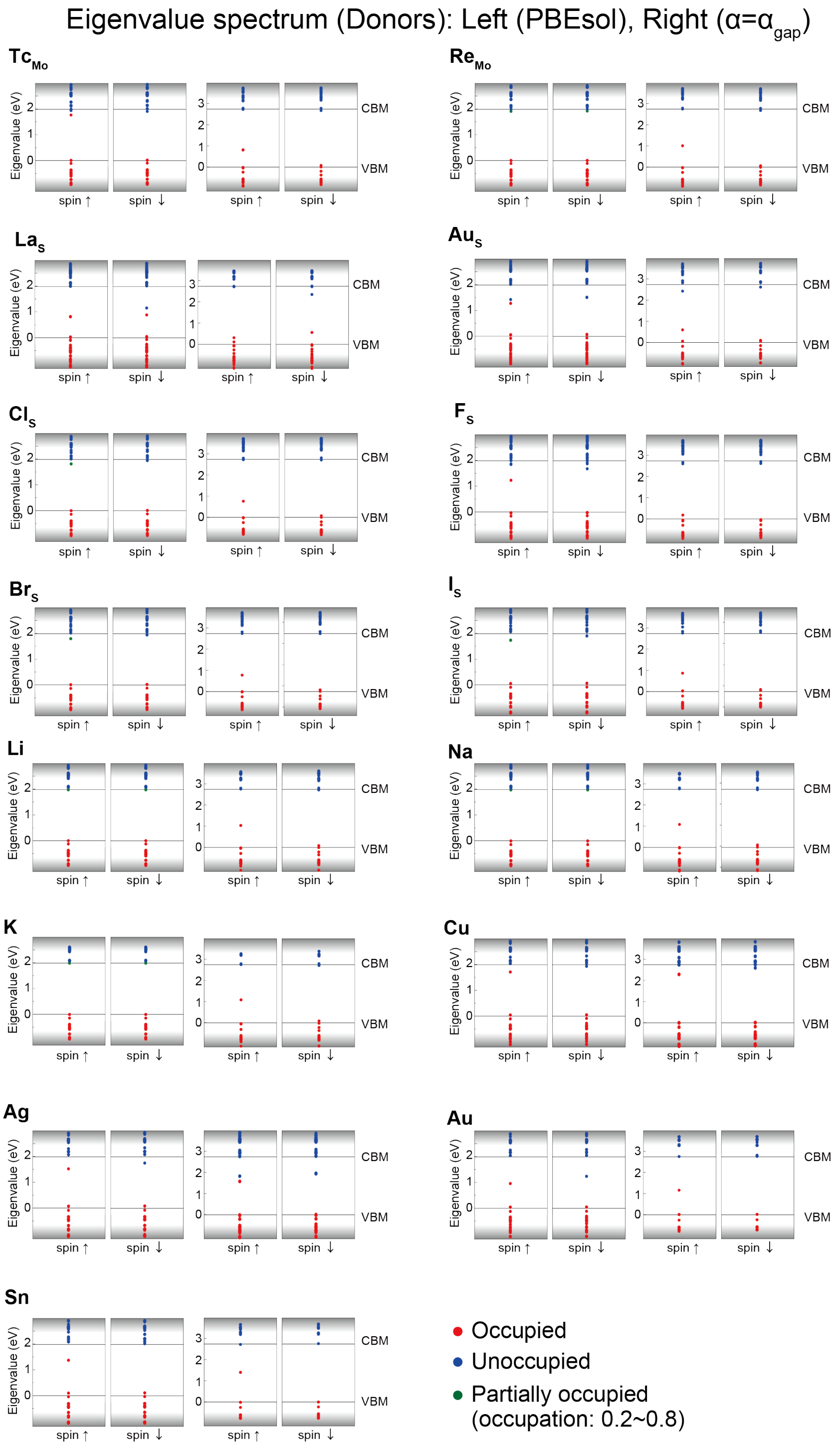

Figure S10. Eigenvalue spectra of 15 types of donors including Mo substitutions (Tc_{Mo}, and Re_{Mo}), S substitutions (La_S, Au_S, Cl_S, F_S, Br_S, and I_S) and surface adsorptions (Li, Na, K, Cu, Ag, Au, and Sn) in the monolayer MoS_2. The eigenvalues and occupations are given at the Γ point of the reciprocal space of the monolayer MoS_2 in the spin up and spin down channels (denoted with spin ↑, and spin ↓). The different electron occupations are denoted by the color of the labels, as explained on the left bottom of the figure.

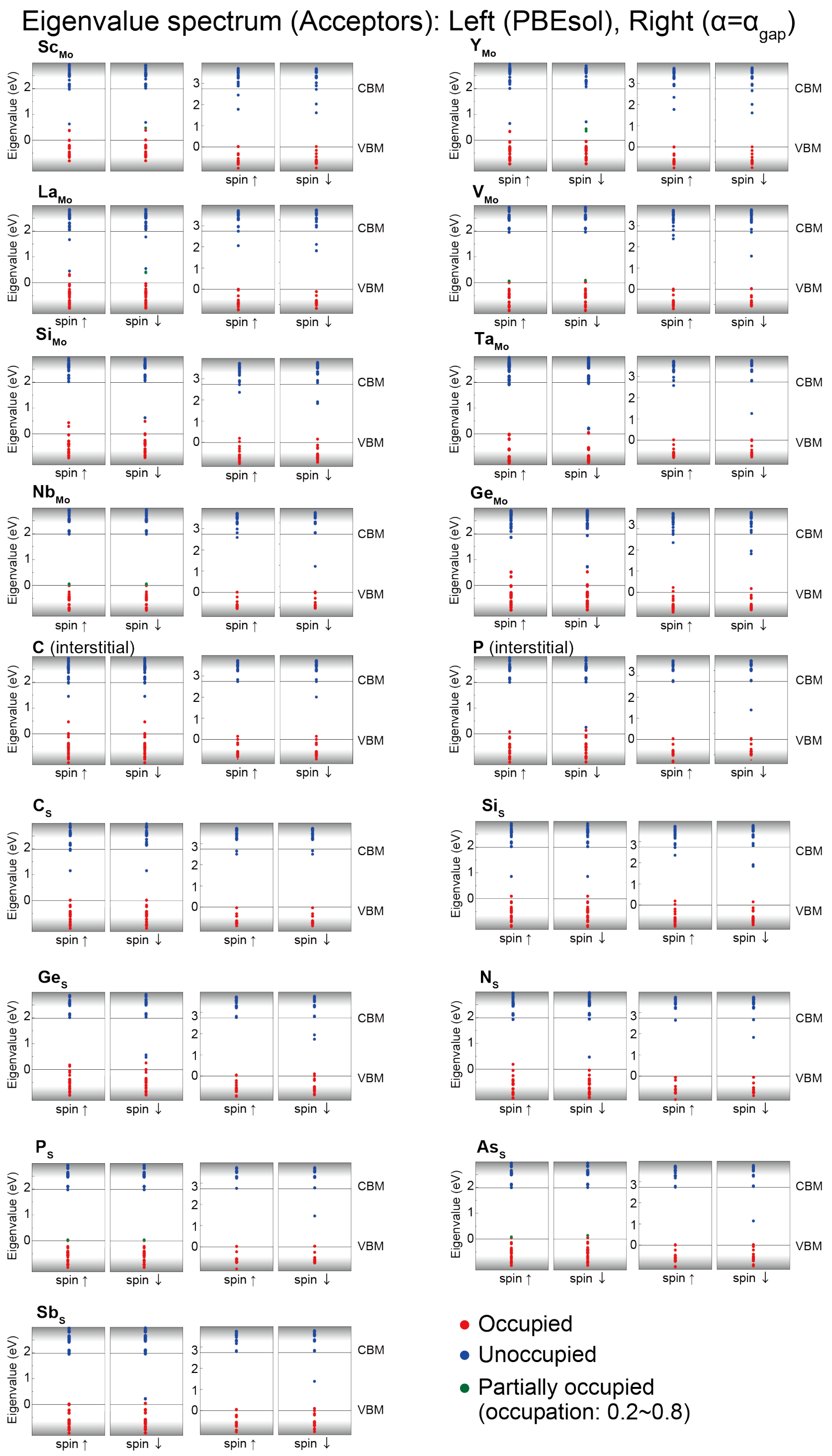

Figure S11. Eigenvalue spectra of 17 types of acceptors including Mo substitutions (Sc_{Mo}, Y_{Mo}, La_{Mo}, V_{Mo}, Si_{Mo}, Ta_{Mo}, Nb_{Mo}, and Ge_{Mo}), lattice interstitials (C, and P), and S substitutions (C_S, Si_S, Ge_S, N_S, P_S, As_S, and Sb_S) in the monolayer MoS_2. The eigenvalues and occupations are given at the Γ point of the reciprocal space of the monolayer MoS_2 in the spin up and spin down channels (denoted with spin ↑, and spin ↓). The different electron occupations are denoted by the color of the labels, as explained on the left bottom of the figure.

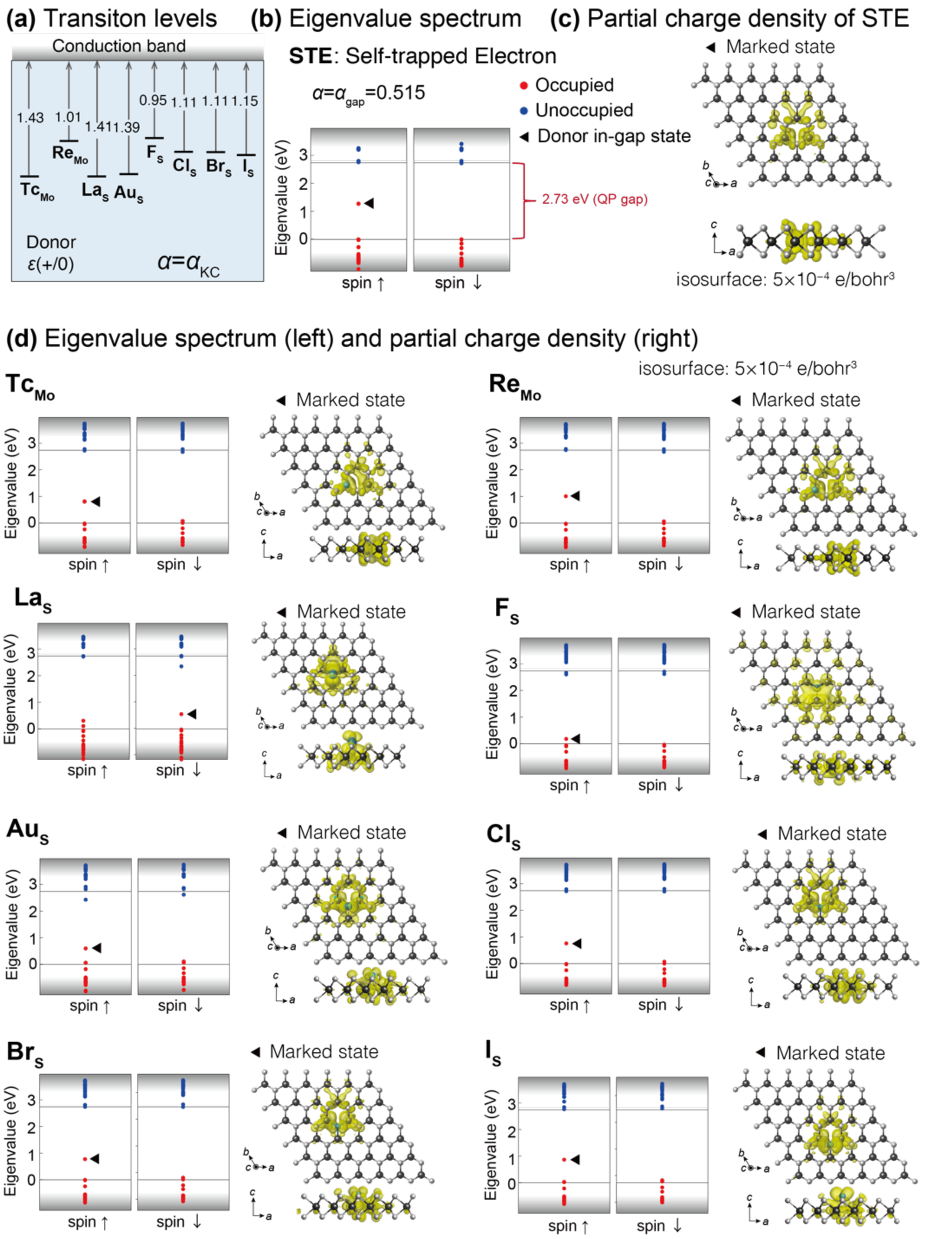

Figure S12. Eigenvalue spectra and partial charge densities of polaronic donor states in the monolayer MoS_2. (a) Donor transition levels $\varepsilon(+/0)$ of 8 donors (Tc_{Mo}, Re_{Mo}, La_S, F_S, Au_S, Cl_S, and Br_S) obtained using the HSE hybrid functional (the exchange screening parameter μ = 0.208 Å^{-1}) with the Koopmans-compliant mixing parameters α_{KC}. (b) The eigenvalue spectrum of the self-trapped electron (STE) in the monolayer MoS_2 where the mixing parameter α_{gap}= 0.515 eV is employed. The STE originated in-gap state is marked in the eigenvalue spectra. (c) The partial charge density plot of the self-trapped electron viewed from the *c* axis (top panel) and *a* axis (bottom panel). The isosurface of the partial charge density is set to 5×10^{-4} e/bohr^3. (d) Eigenvalue spectra (left) and partial charge density plots of the in-gap occupied state (right) of 8 donors. The occupied in-gap states (polaronic donor state) are marked in the eigenvalue spectra, and their partial charge density are shown.

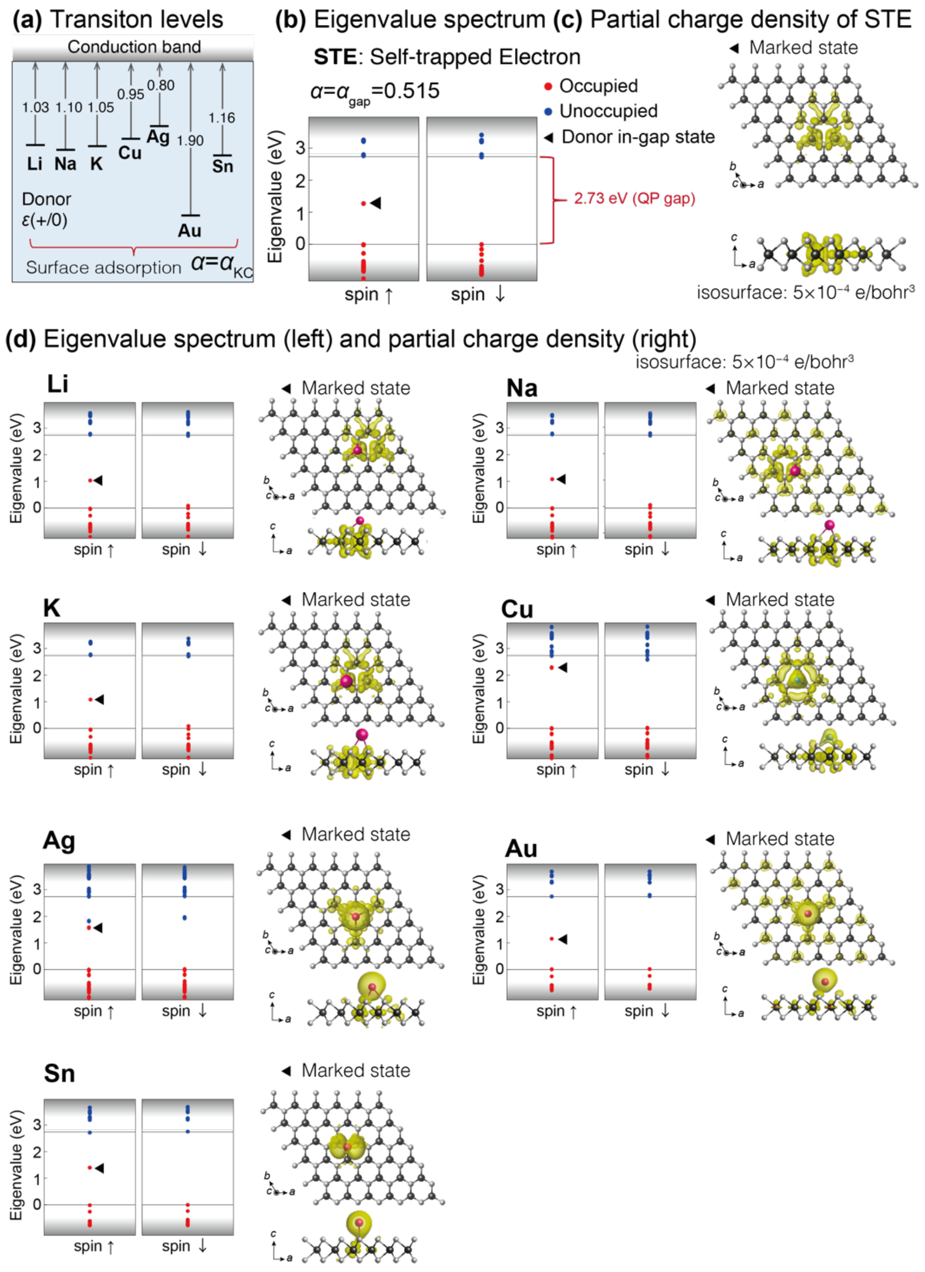

Figure S13. Eigenvalue spectra and partial charge densities of polaronic donor states in the monolayer MoS_2. (a) Donor transition levels $\varepsilon(+/0)$ of 7 donor dopants of surface adsorptions (Li, Na, K, Cu, Ag, Au, and Sn) obtained using the HSE hybrid functional (the exchange screening parameter μ = 0.208 $Å^{-1}$) with the Koopmans-compliant mixing parameters α_{KC}. (b) The eigenvalue spectrum of the self-trapped electron (STE) in the monolayer MoS_2 where the mixing parameter α_{gap}= 0.515 eV is employed. The STE originated in-gap state is marked. (c) The partial charge density plot of the self-trapped electron viewed from the *c* axis (top panel) and *a* axis (bottom panel). The isosurface of the partial charge density is set to 5×10^{-4} e/bohr^3. (d) Eigenvalue spectra (left) and partial charge density plots of the in-gap occupied state (right) of 7 donor dopants. The occupied in-gap states (polaronic donor state) are marked in the eigenvalue spectra, and their partial charge density are shown.

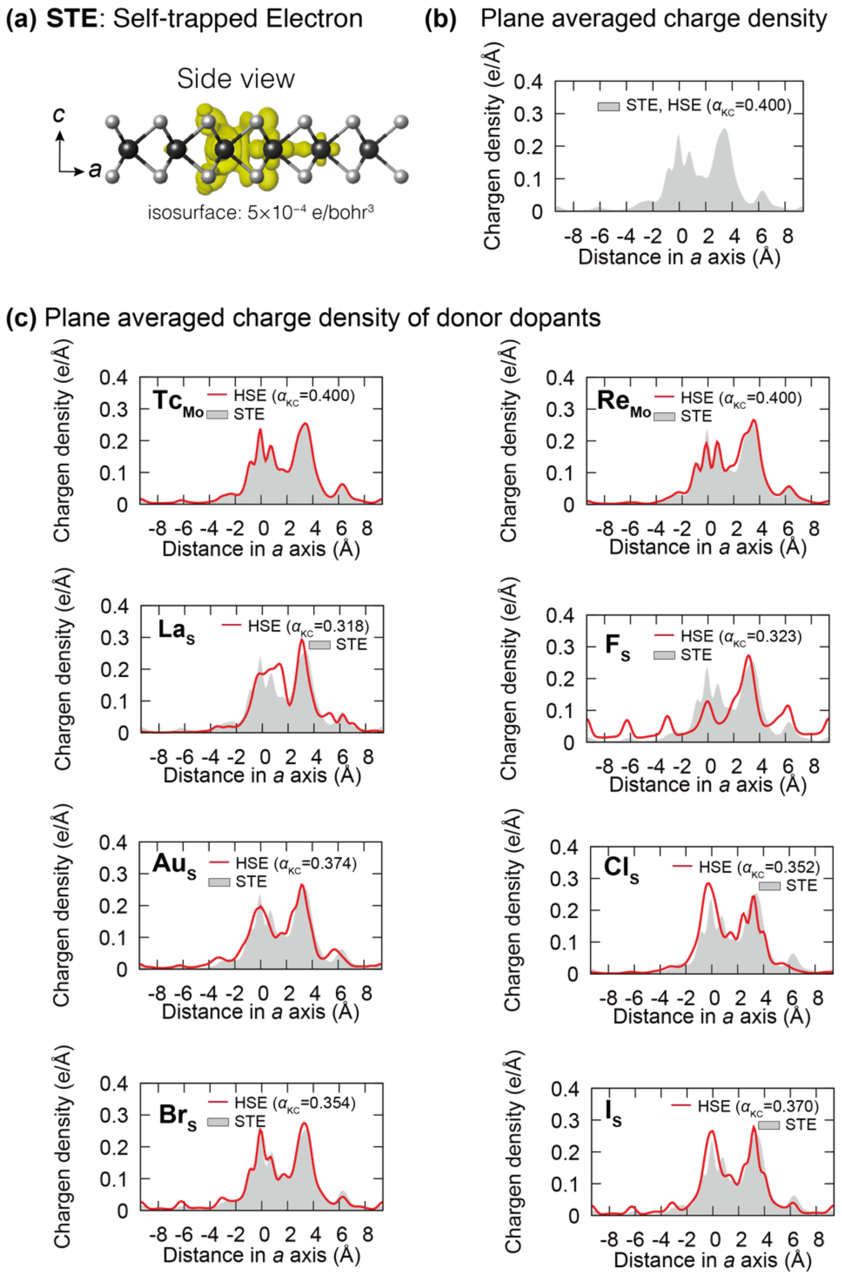

Figure S14. Plane averaged partial charge densities of the self-trapped electron (STE) and the polaronic donor states in the monolayer MoS_2. (a) A side view of the partial charge density of the self-trapped electron obtained with the HSE functional of the Koopmans compliant Fock exchange mixing parameter $\alpha=\alpha_{KC}$ (= 0.400 for STE). (b) The plane averaged of the partial charge density of the self-trapped electron (STE) along *a* axis. (c) Plane averaged charge densities of the occupied in-gap states of 7 donors obtained with the HSE functional of the Koopmans compliant Fock exchange mixing parameter $\alpha=\alpha_{KC}$ compared to the STE's plane averaged charge density. The plane averaged charge density of the self-trapped electron (STE) is shown with the filled curves.

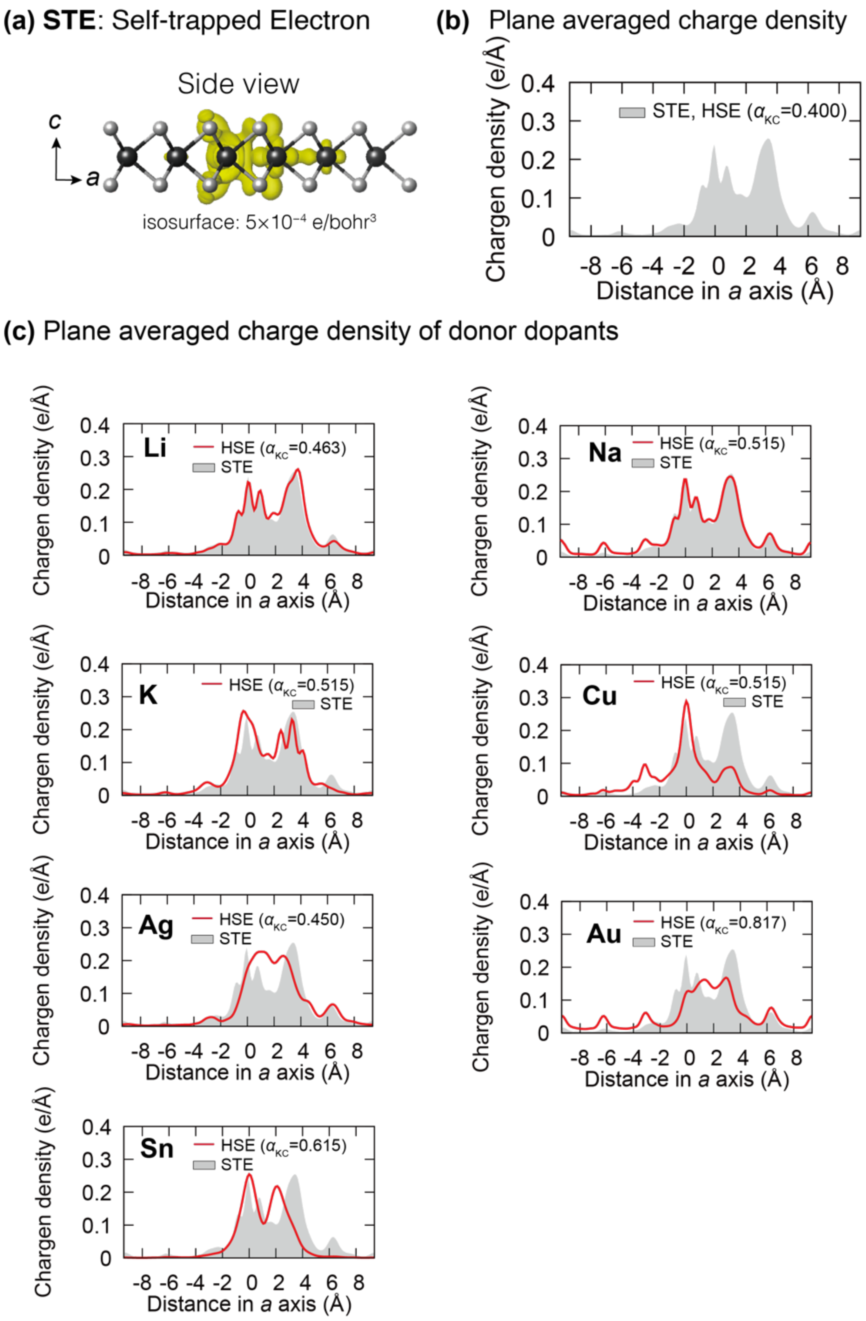

Figure S15. Plane averaged partial charge densities of the self-trapped electron (STE) and the polaronic donor states in the monolayer MoS_2. (a) A side view of the partial charge density of the self-trapped electron obtained with the HSE functional of the Koopmans compliant Fock exchange mixing parameter $\alpha=\alpha_{KC}$ (= 0.400 for STE). (b) The plane averaged of the partial charge density of the self-trapped electron (STE) along *a* axis. (c) Plane averaged charge densities of the occupied in-gap states of 7 donors obtained with the HSE functional of the Koopmans compliant Fock exchange mixing parameter $\alpha=\alpha_{KC}$ compared to the STE's plane averaged charge density. The plane averaged charge density of the self-trapped electron (STE) is shown with the filled curves.

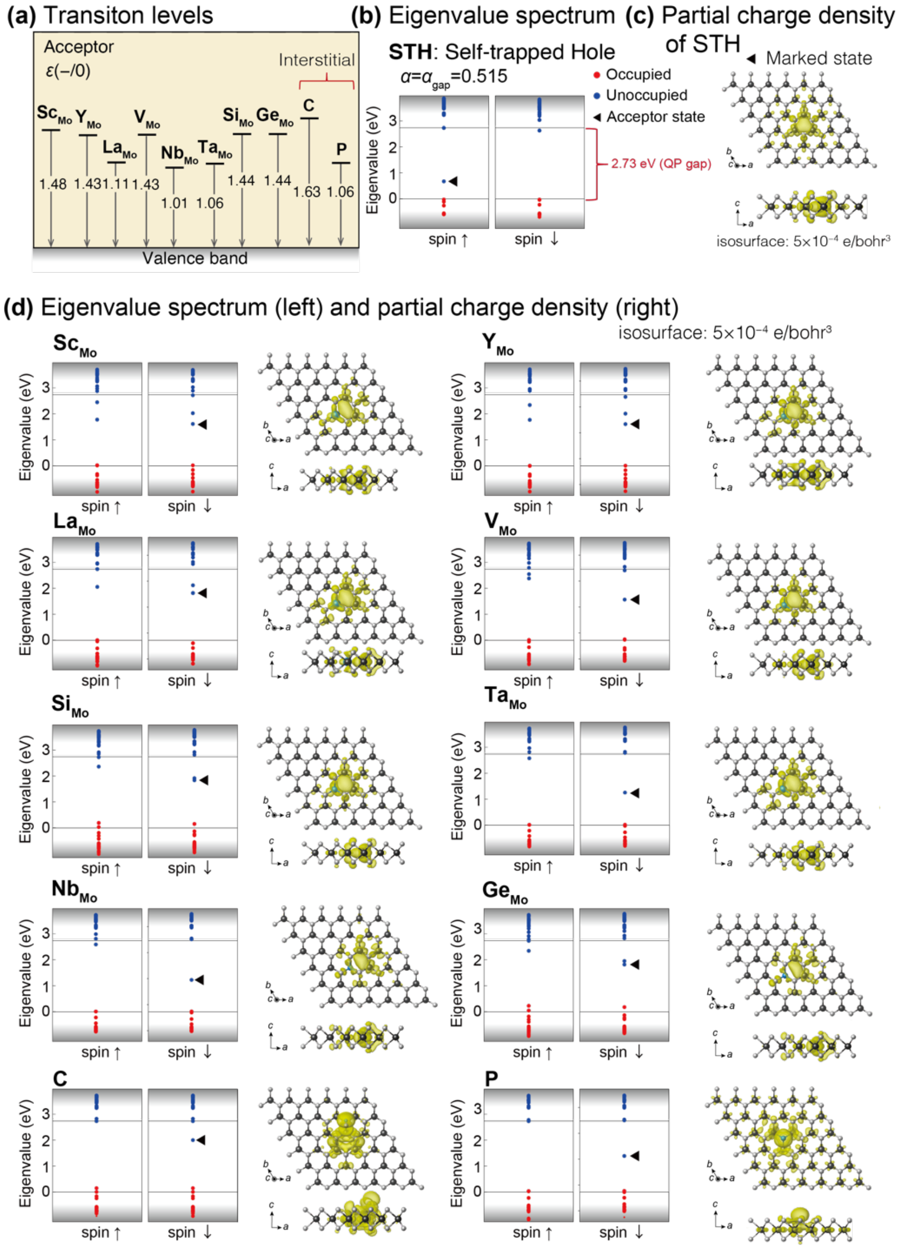

Figure S16. Eigenvalue spectra and partial charge densities of polaronic acceptor states. (a) Acceptor transition levels [$\varepsilon(-/0)$] of 10 acceptors including Mo substitutions (Sc_{Mo}, Y_{Mo}, La_{Mo}, V_{Mo}, Si_{Mo}, Ta_{Mo}, Y_{Mo}, Nb_{Mo}, and Ge_{Mo}) and lattice interstitials (C and P) obtained using the HSE hybrid functional (the exchange screening parameter μ = 0.208 Å^{-1}) with the Koopmans-compliant mixing parameters α_{KC}. (b) The eigenvalue spectrum of the self-trapped hole (STE) in the monolayer MoS_2 where the mixing parameter α_{gap}= 0.515 eV is adopted. The STH originated in-gap state is marked. (c) The partial charge density plot of the self-trapped electron viewed from the *c* axis (top panel) and *a* axis (bottom panel). The isosurface of the partial charge density is set to 5×10^{-4} e/bohr^3. (d) Eigenvalue spectra (left) and partial charge density plots of the in-gap unoccupied state (right) of 10 acceptors. The unoccupied in-gap states (polaronic acceptor state) are marked, and their partial charge density are visualized.

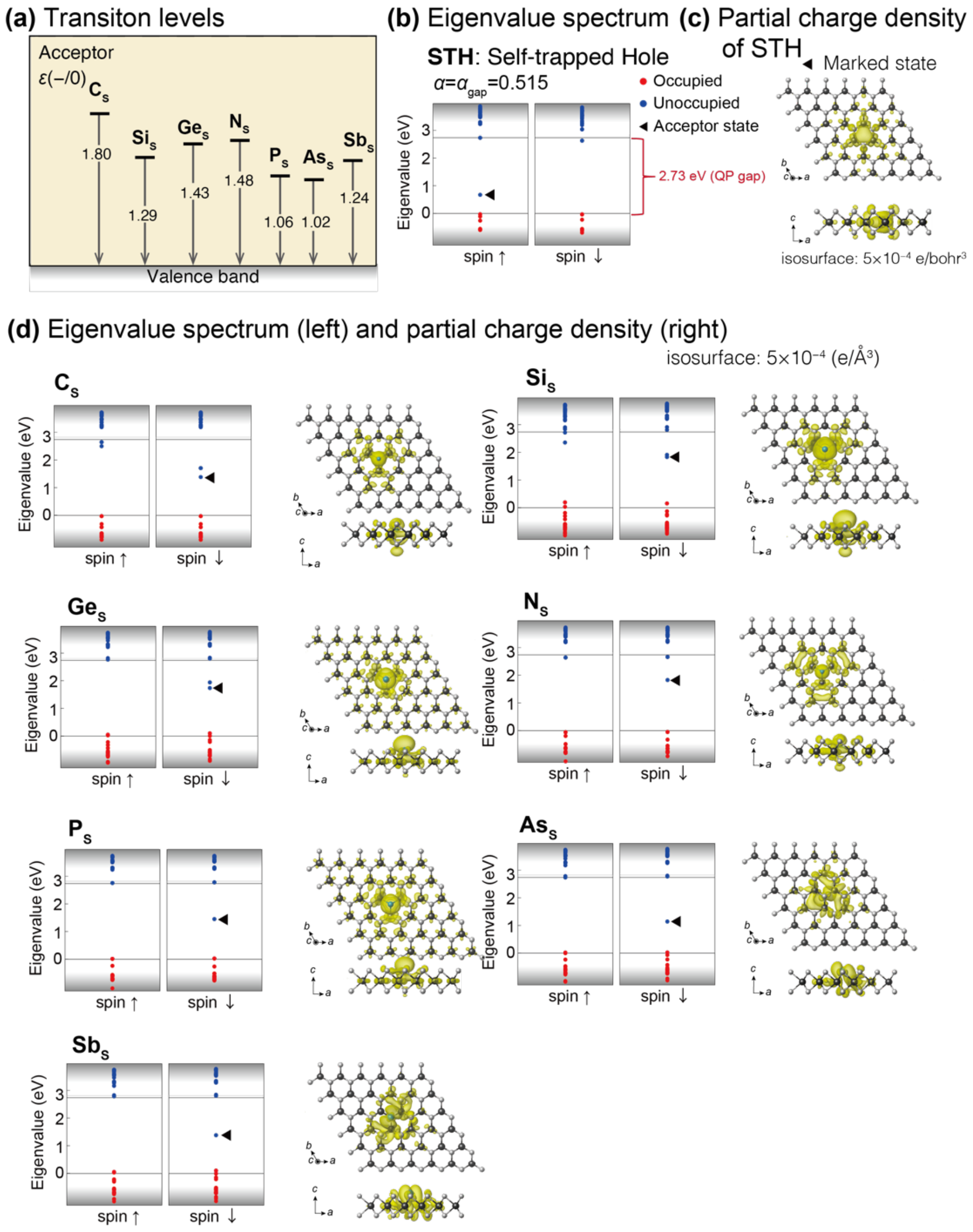

Figure S17. Eigenvalue spectra and partial charge densities of polaronic acceptor states. (a) Acceptor transition levels [$\varepsilon(-/0)$] of 7 acceptors of S substitutions (C_S, Si_S, Ge_S, N_S, P_S, As_S, and Sb_S) obtained using the HSE hybrid functional (the exchange screening parameter μ = 0.208 Å^{-1}) with the Koopmans-compliant mixing parameters α_{KC}. (b) The eigenvalue spectrum of the self-trapped hole (STE) in the monolayer MoS_2 where the mixing parameter α_{gap}= 0.515 eV is adopted. The STH originated in-gap state is marked. (c) The partial charge density plot of the self-trapped electron viewed from the *c* axis (top panel) and *a* axis (bottom panel). The isosurface of the partial charge density is set to 5×10^{-4} e/bohr^3. (d) Eigenvalue spectra (left) and partial charge density plots of the in-gap unoccupied state (right) of 8 acceptors. The unoccupied in-gap states (polaronic acceptor state) are marked, and their partial charge density are visualized.

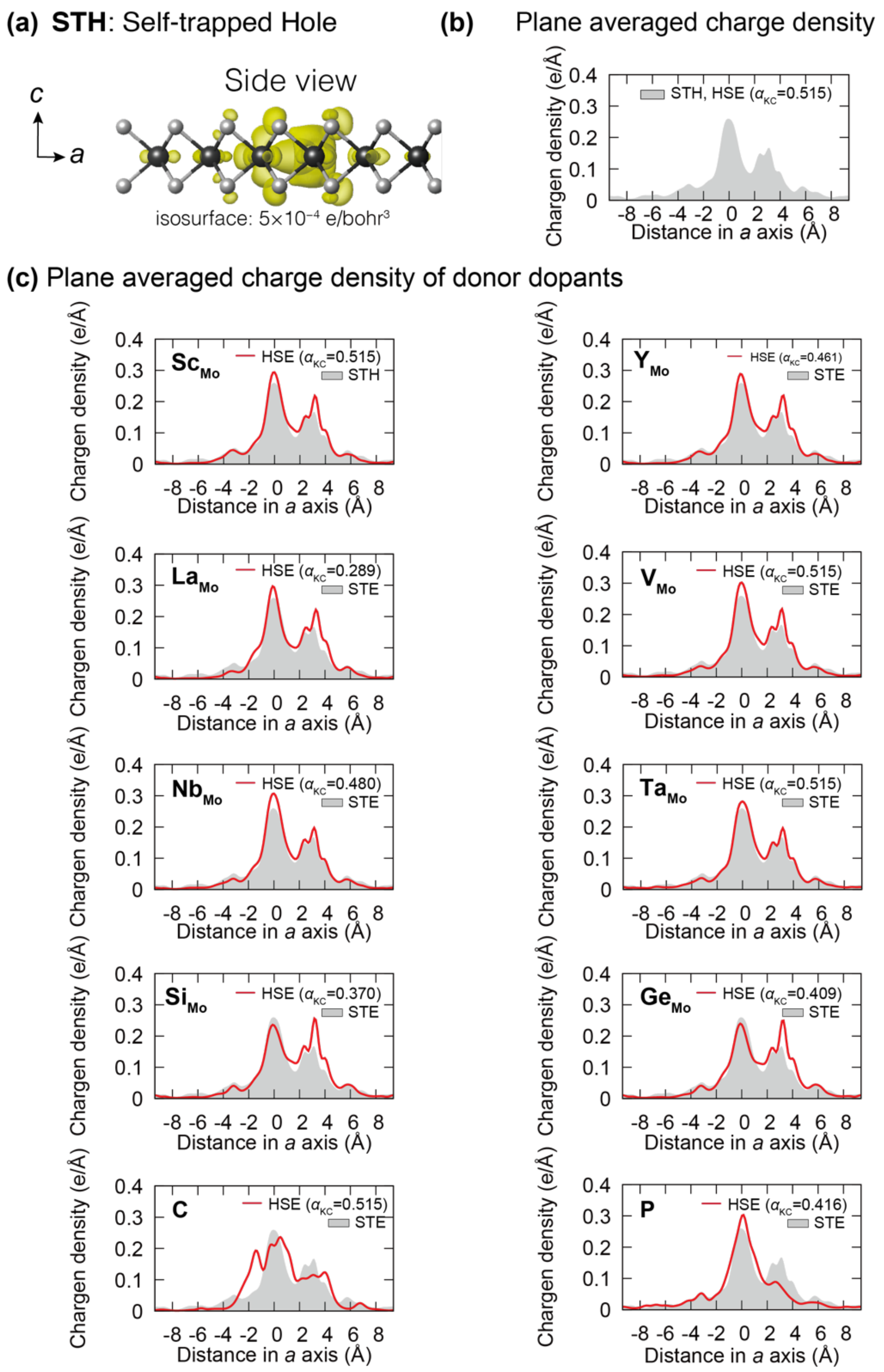

Figure S18. Plane averaged partial charge densities of the self-trapped hole (STH) and the polaronic acceptor states in the monolayer MoS_2. (a) A side view of the partial charge density of the self-trapped electron obtained with the HSE functional of the Koopmans compliant Fock exchange mixing parameter α=α_{KC} (= 0.515 for STH). (b) The plane averaged of the partial charge density of the self-trapped hole (STH) along *a* axis. (c) Plane averaged charge densities of the occupied in-gap states of 10 acceptors obtained with the HSE functional of the Koopmans compliant Fock exchange mixing parameter α=α_{KC} compared to the STH's plane averaged charge density. The plane averaged charge density of the self-trapped hole (STH) is shown with the filled curves.

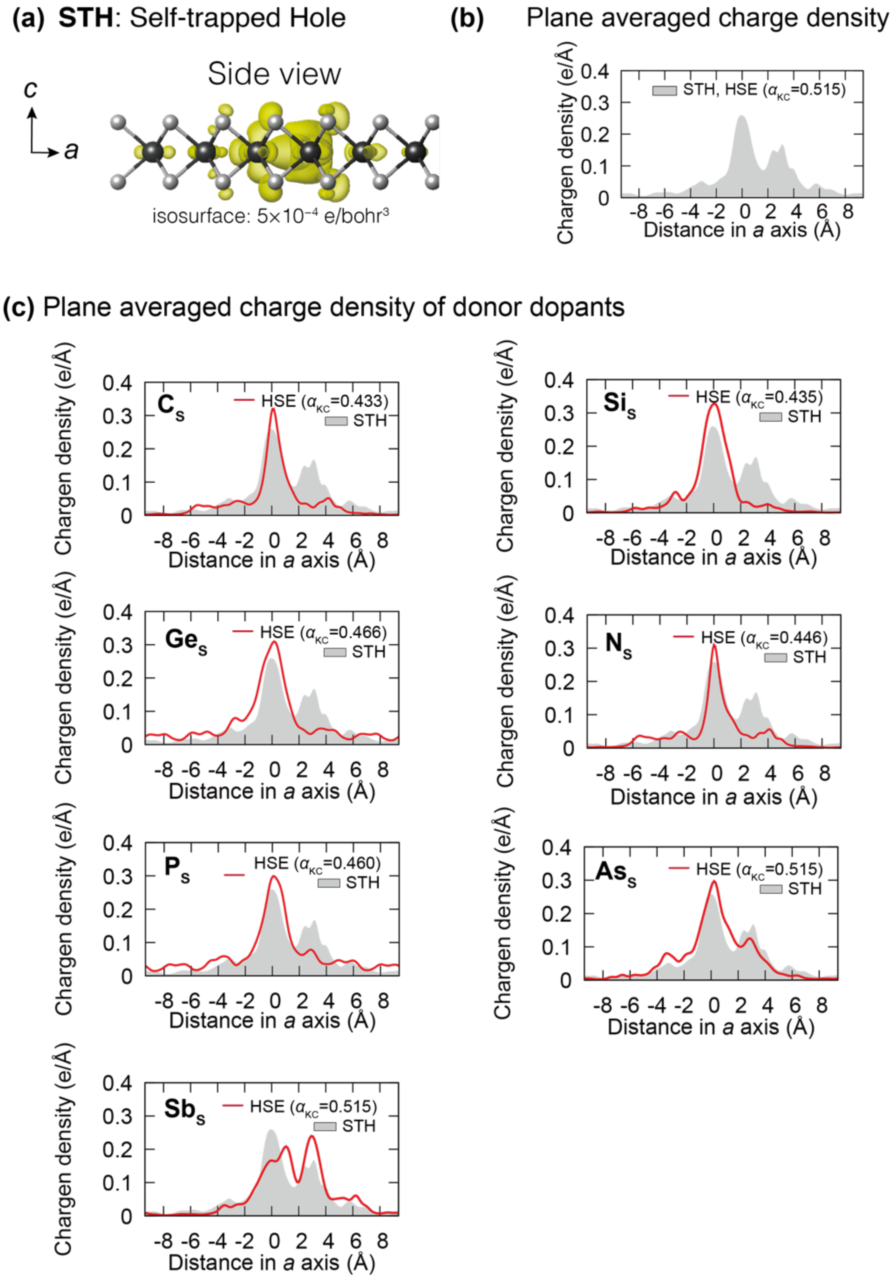

Figure S19. Plane averaged partial charge densities of the self-trapped hole (STH) and the polaronic acceptor states in the monolayer MoS_2. (a) A side view of the partial charge density of the self-trapped electron obtained with the HSE functional of the Koopmans compliant Fock exchange mixing parameter $\alpha=\alpha_{KC}$ (= 0.515 for STH). (b) The plane averaged of the partial charge density of the self-trapped hole (STH) along *a* axis. (c) Plane averaged charge densities of the occupied in-gap states of 7 acceptors obtained with the HSE functional of the Koopmans compliant Fock exchange mixing parameter $\alpha=\alpha_{KC}$ compared to the STH's plane averaged charge density. The plane averaged charge density of the self-trapped hole (STH) is shown with the filled curves.

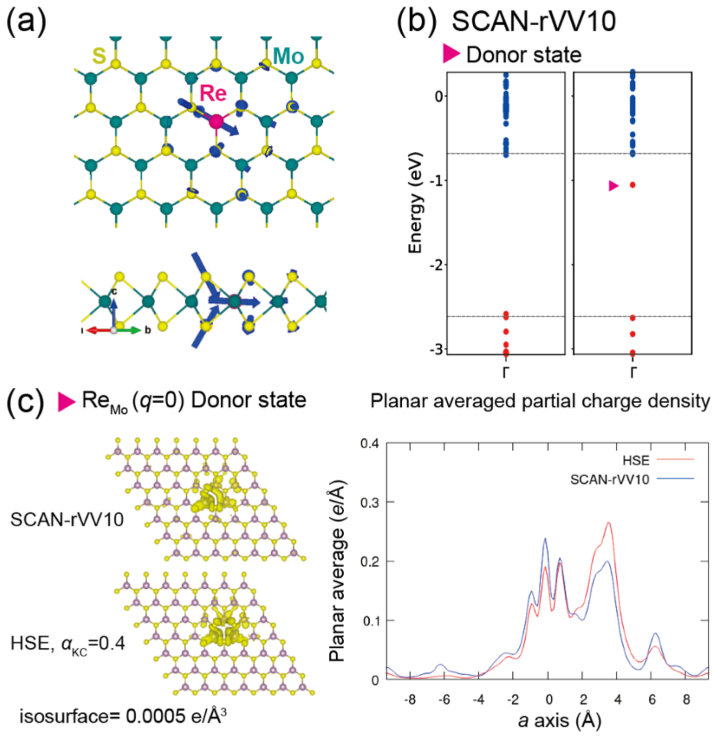

Figure S20. Comparison of Re_{Mo} (q=0) results obtained with SCAN+rVV10 and HSE functionals (a) A top view and side view of atomic distortion from the symmetric to symmetry-broken polaronic state obtained with the SCAN+rVV10 functional. (b) The eigenvalue plot of the polaronic state obtained with the SCAN+rVV10 functional. (c) Partial charge densities of the polaronic Re_{Mo} (q=0) donor state obtained with the SCAN+rVV10 and HSE (α_{KC} = 0.4) functionals. The isosurface of the partial charge density plots is set to 0.0005 e/Å^3 and their planar averages are plotted along the *a* axis.

Table S1. Chemical potentials at Mo-rich and S-rich condition. Chemical potentials of dopant μ_{Dopant} (in eV), impurity phases at the Mo-rich and S-rich conditions obtained with the PBEsol functional are listed. At the Mo-rich condition, $\mu_{Mo} = 0.00$ eV and $\mu_S = -1.42$ eV competing with the bcc Mo crystal. At the S-rich condition, $\mu_{Mo} =-2.83$ eV and $\mu_S = 0.00$ eV competing with the orthorhombic S crystal.

	Mo-rich		S-rich	
Dopant	μ_{Dopant}	Impurity phase	μ_{Dopant}	Impurity phase
**Li**	-1.63	$Li_2Mo_3S_4$	-2.02	Li_2S
**Na**	-1.42	$Na_{17}(Mo_{15}S_{19})_4$	-1.83	NaS_2
**K**	-1.56	$K(MoS)_3$	-2.11	KS_3
**Sc**	-2.77	Sc_2S_3	-4.89	Sc_2S_3
**Y**	-3.21	Y_2S_3	-5.33	Y_2S_3
**La**	-3.84	$La(Mo_3S_4)_2$	-5.80	LaS_2
**V**	-0.70	V_5S_4	-2.90	VS_4
**Nb**	-0.89	Nb_3S4	-3.46	NbS_3
**Ta**	-0.70	Ta_5S_8	-3.53	TaS_3
**Mn**	0.00	Mn	-0.70	MnS_2
**Tc**	0.00	Tc	-2.07	TcS_2
**Re**	0.00	Re	-1.89	ReS_2
**Cu**	0.00	Cu	-0.45	CuS
**Ag**	0.00	Ag	-0.04	Ag_2S
**Au**	0.00	Au	0.00	Au
**C**	-0.47	Mo_2C	0.00	C
**Si**	-1.49	$SiMo_3$	-1.96	SiS_2
**Ge**	-0.79	$GeMo_3$	-0.95	GeS_2
**Sn**	0.00	Sn	-1.33	SnS_2
**N**	-1.26	MoN	0.00	N
**P**	-1.63	Mo_3P	-0.62	P_2S_7
**As**	-0.70	Mo_5As_4	-0.26	As_2S_3
**Sb**	-0.11	$Sb(MoS)_2$	-0.70	Sb_2S_3
**F**	-4.68	MoF_6	-4.21	MoF_6
**Br**	-1.13	Mo_2SBr_2	-0.06	SBr
**Cl**	-1.44	$MoCl_2$	-0.48	$Mo_3S_7Cl_4$
**I**	-0.59	MoI_2	0.00	I

Table S2. Defect formation energy of donor dopants at the Mo-rich and S-rich conditions and their difference between the PBEsol and HSE results. In calculations, the semi-local PBEsol functional and the HSE functional (μ = 0.208 Å^{-1} and α= α_{gap}= 0.515) are employed. The difference in defect formation energy is evaluated from ΔE_f(PBEsol)−ΔE_f(HSE), and the dopants with a difference larger than 0.4 eV are highlighted in bold, which denotes that they are predicted as two-dimensional hydrogenic states with the PBEsol functional, while they are consistently localized into a small polaronic state with the HSE functional (the *shallow-to-deep transition* in Fig. S5).

	ΔE_f (eV) (q =0), Mo-rich PBEsol	ΔE_f (eV) (q =0), S-rich PBEsol	ΔE_f (eV) (q =0), Mo-rich HSE (α_{gap}= 0.515)	ΔE_f (eV) (q =0), S-rich HSE (α_{gap}= 0.515)	ΔE_f(PBEsol)−ΔE_f (HSE,α_{gap}= 0.515)
**Tc_{Mo}**	1.68	0.92	1.21	0.44	**0.48**
**Re_{Mo}**	1.93	0.99	1.45	0.50	**0.49**
La_S	3.18	6.56	3.07	6.45	0.12
Au_S	2.07	3.49	1.89	3.31	0.19
F_S	1.14	2.08	0.83	1.77	0.30
Cl_S	1.43	1.89	1.13	1.59	0.30
Br_S	1.57	1.92	1.18	1.53	0.39
I_S	1.52	2.35	1.21	2.05	0.30
**$Li_{surface}$**	1.58	1.97	1.09	1.48	**0.49**
**$Na_{surface}$**	0.85	1.75	0.39	1.29	**0.46**
**$K_{surface}$**	0.85	1.40	0.38	0.93	**0.46**
$Cu_{surface}$	2.26	2.71	2.07	2.52	0.18
$Ag_{surface}$	2.26	2.30	2.16	2.20	0.10
$Au_{surface}$	2.71	2.71	2.57	2.57	0.15
$Sn_{surface}$	2.35	3.68	2.58	3.91	-0.23

Table S3. Defect formation energy of acceptor dopants at the Mo-rich and S-rich conditions and their difference between the PBEsol and HSE results. In calculations, the semi-local PBEsol functional and the HSE functional (μ = 0.208 Å^{-1} and α= α_{gap}= 0.515) are employed. The difference in defect formation energy is evaluated from ΔE_f(PBEsol)−ΔE_f(HSE), and the dopants with a difference larger than 0.4 eV are highlighted in bold, which denotes that they are predicted as two-dimensional hydrogenic states with the PBEsol functional, while they are consistently localized into a small polaronic state with the HSE functional (the *shallow-to-deep transition* in Fig. S6).

	ΔE_f (eV) (q =0), Mo-rich PBEsol	ΔE_f (eV) (q =0), S-rich PBEsol	ΔE_f (eV) (q =0), Mo-rich HSE (α_{gap}= 0.515)	ΔE_f (eV) (q =0), S-rich HSE (α_{gap}= 0.515)	ΔE_f(PBEsol)−ΔEf (HSE,α_{gap}= 0.515)
Sc_{Mo}	3.60	2.89	3.45	2.74	0.16
Y_{Mo}	4.73	4.02	4.58	3.87	0.15
La_{Mo}	6.92	6.05	6.77	5.90	0.15
**V_{Mo}**	1.09	0.46	0.58	-0.05	**0.51**
**Nb_{Mo}**	0.83	0.57	0.30	0.04	**0.53**
**Ta_{Mo}**	0.52	0.52	0.08	0.08	**0.44**
Si_{Mo}	5.74	3.38	5.95	3.59	-0.21
Ge_{Mo}	6.10	3.43	6.24	3.57	-0.14
$C_{interstitial}$	3.70	3.23	3.90	3.43	-0.20
$P_{interstitial}$	3.17	2.16	3.30	2.29	-0.12
C_S	2.98	3.92	3.07	4.01	-0.09
Si_S	3.67	5.56	3.84	5.73	-0.17
Ge_S	2.91	4.49	2.99	4.57	-0.07
N_S	1.66	1.81	1.82	1.97	-0.16
P_S	2.38	2.79	2.38	2.79	0.00
As_S	1.43	2.41	1.46	2.44	-0.02
Sb_S	1.10	3.11	1.14	3.15	-0.04

Table S4. Koopmans-compliant mixing parameter (α_{KC}), the transition levels [ε(+/0)] of donor dopants obtained with the PBEsol functional and HSE functionals with Fock exchange mixing parameters of α_{gap}= 0.515 and α_{KC}, and carrier–dopant binding energy of polaronic donors ($E_{b,donor}$). The screening parameter of the HSE hybrid functional is fixed μ = 0.208 Å^{-1}. The non-Koopmans energy ΔE_{NK} is defined as $\Delta E_{NK}= \varepsilon_i - [E(q=0)-E(q=+1)]$, where ε_i is the eigenvalue of the polaronic donor state and $E(q=0)$ and $E(q=+1)$ are the total energy of q=0 (neutral), and +1 (ionized) acceptor states. The definition of the carrier-dopant binding energy is provided in the **METHODS** section.

	α_{KC}	Non-Koopmans' energy ($\alpha=\alpha_{gap}$=0.515)	ε(+/0) (PBEsol)	ε(+/0) ($\alpha=\alpha_{gap}$=0.515)	ε(+/0) ($\alpha=\alpha_{KC}$)	$E_{b,donor}$ (eV)
Tc_{Mo}	0.400	-0.21	0.78	1.44	1.43	-1.65
Re_{Mo}	0.400	0.24	0.65	1.19	1.01	-1.40
La_S	0.318	0.31	1.39	1.78	1.41	-1.95
Au_S	0.374	0.30	1.21	1.80	1.39	-2.00
F_S	0.323	0.41	1.01	1.05	0.95	-1.89
Cl_S	0.352	0.35	0.72	1.42	1.10	-1.68
Br_S	0.354	-0.34	0.75	1.41	1.11	-1.62
I_S	0.370	0.29	0.79	1.40	1.15	-1.61
$Li_{surface}$	0.463	-0.11	0.62	1.04	1.03	-1.36
$Na_{surface}$	0.515	0.03	0.61	1.10	1.10	-1.31
$K_{surface}$	0.515	-0.03	0.63	1.05	1.05	-1.26
$Cu_{surface}$	0.515	-0.03	0.80	0.95	0.95	-1.16
$Ag_{surface}$	0.450	0.25	1.01	0.91	0.80	-1.12
$Au_{surface}$	0.817	0.22	1.72	1.77	1.90	-1.98
$Sn_{surface}$	0.615	0.17	0.82	1.08	1.16	-1.31

Table S5. Koopmans-compliant mixing parameter (α_{KC}), the transition levels [ε(+/0)] of acceptor dopants obtained with the PBEsol functional and HSE functionals with Fock exchange mixing parameters of α_{gap}= 0.515 and α_{KC}, and carrier–dopant binding energy of polaronic acceptors ($E_{b,acceptor}$). The screening parameter of the HSE hybrid functional is fixed μ = 0.208 Å^{-1}. The non-Koopmans energy ΔE_{NK} is defined as $\Delta E_{NK}= \varepsilon_i - [E(q=-1)-E(q=0)]$, where ε_i is the eigenvalue of the polaronic donor state and $E(q=-1)$ and $E(q=0)$ are the total energy of q=–1 (ionized), and 0 (neutral) acceptor states. The definition of the carrier-dopant binding energy is provided in the **METHODS** section.

	α_{KC}	Non-Koopmans' energy ($\alpha=\alpha_{gap}$=0.515)	ε(+/0) (PBEsol)	ε(+/0) ($\alpha=\alpha_{gap}$=0.515)	ε(+/0) ($\alpha=\alpha_{KC}$)	$E_{b,acceptor}$ (eV)
Sc_{Mo}	0.515	0.01	0.81	1.48	1.48	-1.92
Y_{Mo}	0.461	-0.09	0.79	1.48	1.43	-1.91
La_{Mo}	0.289	0.67	0.78	1.52	1.11	-1.96
V_{Mo}	0.515	-0.20	0.63	1.43	1.43	-1.86
Nb_{Mo}	0.480	0.05	0.61	1.04	1.01	-1.48
Ta_{Mo}	0.515	-0.03	0.62	1.06	1.06	-1.50
Si_{Mo}	0.370	-0.32	0.99	1.52	1.44	-1.92
Ge_{Mo}	0.409	-0.22	1.04	1.54	1.44	-1.97
$C_{interstitial}$	0.515	0.04	1.29	1.63	1.63	-2.07
$P_{interstitial}$	0.416	-0.15	0.71	1.12	1.06	-1.55
C_S	0.433	-0.12	1.46	1.84	1.80	-2.28
Si_S	0.435	-0.14	0.99	1.33	1.29	-1.76
Ge_S	0.466	-0.07	0.96	1.47	1.43	-1.90
N_S	0.446	-0.16	0.99	1.57	1.48	-1.99
P_S	0.460	-0.09	0.61	1.09	1.06	-1.53
As_S	0.515	-0.02	0.67	1.02	1.02	-1.45
Sb_S	0.515	0.02	0.67	1.24	1.24	-1.68
